# mRNA decoding in human is kinetically and structurally distinct from bacteria

**DOI:** 10.1038/s41586-023-05908-w

**Published:** 2023-04-05

**Authors:** Mikael Holm, S. Kundhavai Natchiar, Emily J. Rundlet, Alexander G. Myasnikov, Zoe L. Watson, Roger B. Altman, Hao-Yuan Wang, Jack Taunton, Scott C. Blanchard

**Affiliations:** 1 Department of Structural Biology, St Jude Children’s Research Hospital, Memphis, TN USA; 2 Tri-Institutional PhD Program in Chemical Biology, Weill Cornell Medicine, New York, NY USA; 3 California Institute for Quantitative Biosciences, University of California, Berkeley, CA USA; 4 Department of Cellular and Molecular Pharmacology, University of California, San Francisco, CA USA; 5 Chemical Biology & Therapeutics, St Jude Children’s Research Hospital, Memphis, TN USA; 6 Present Address: Dubochet Center for Imaging (DCI), EPFL, Lausanne, Switzerland

**Keywords:** Single-molecule biophysics, Ribosome, Cryoelectron microscopy

## Abstract

In all species, ribosomes synthesize proteins by faithfully decoding messenger RNA (mRNA) nucleotide sequences using aminoacyl-tRNA substrates. Current knowledge of the decoding mechanism derives principally from studies on bacterial systems^1^. Although key features are conserved across evolution^2^, eukaryotes achieve higher-fidelity mRNA decoding than bacteria^3^. In human, changes in decoding fidelity are linked to ageing and disease and represent a potential point of therapeutic intervention in both viral and cancer treatment^4–6^. Here we combine single-molecule imaging and cryogenic electron microscopy methods to examine the molecular basis of human ribosome fidelity to reveal that the decoding mechanism is both kinetically and structurally distinct from that of bacteria. Although decoding is globally analogous in both species, the reaction coordinate of aminoacyl-tRNA movement is altered on the human ribosome and the process is an order of magnitude slower. These distinctions arise from eukaryote-specific structural elements in the human ribosome and in the elongation factor eukaryotic elongation factor 1A (eEF1A) that together coordinate faithful tRNA incorporation at each mRNA codon. The distinct nature and timing of conformational changes within the ribosome and eEF1A rationalize how increased decoding fidelity is achieved and potentially regulated in eukaryotic species.

## Abstract (web summary)

The reaction coordinate of aminoacyl-tRNA movement is altered on the human ribosome and the process is an order of magnitude slower compared with bacteria due to eukaryote-specific structural elements in the human ribosome and in the elongation factor eEF1A.

## Main

The genetic code that translates mRNA to protein sequence is established by the two-subunit ribosome—a multi-megadalton RNA–protein assembly. The core regions of the large (LSU) and small (SSU) ribosomal subunits are evolutionary conserved across species^2^. This conservation reflects the ubiquitous demand for ribosomes to rapidly and accurately interact with structurally similar, yet sequence-diverse, aminoacyl-tRNA (aa-tRNA) adaptor molecules^1^. In human, emerging therapies target the mRNA-decoding process to treat monogenic diseases^4^, viral infections^5^ and cancer^6^. Structural and regulatory differences across species also underpin antibiotic efficacy^7^.

Extensive biochemical, kinetic and structural investigations principally performed in bacteria^1,8–11^ have revealed that decoding hinges on a two-step kinetic proofreading mechanism. In bacteria, decoding begins with initial selection, in which aa-tRNAs in a ternary complex with GTP and a conserved three-domain GTPase—elongation factor-thermal unstable (EF-Tu)—sample the mRNA codon within the aminoacyl site (A site) at the leading (3′ mRNA) edge of the ribosome. Base pairing between the mRNA codon and the aa-tRNA anticodon stem loop (ASL) is verified through a network of ribosomal RNA (rRNA) and protein interactions within the SSU A site known as the decoding centre. Recognition of cognate aa-tRNA closes the SSU shoulder domain towards the SSU body and head domains. Consequent ternary complex engagement of the LSU GTPase-activating centre (GAC), including the catalytic sarcin-ricin loop^12^ (SRL), induces rearrangements in the GTPase, including switch-I and switch-II remodelling, that trigger GTP hydrolysis^8,12–14^.

GTP hydrolysis initiates the second proofreading selection, during which GTPase remodelling enables the accommodation of the amino-acid-conjugated 3′-CCA end of aa-tRNA into the LSU peptidyl transferase centre (PTC). There, peptide bond formation transfers the nascent peptide chain from the tRNA within the peptidyl-tRNA-binding site (P site) to aa-tRNA. Peptide bond formation terminates decoding, creating the pre-translocation (PRE) complex. Decoding fidelity is established by preferential rejection of incorrect aa-tRNAs during initial selection before GTP hydrolysis and again during proofreading selection before peptide bond formation.

Structural snapshots of mammalian ribosomes isolated from cellular extracts, as well as recent tomographic studies, have provided transformative insights into the mechanistic distinctions of mammalian translation^15–17^. Although the eukaryotic homologue of EF-Tu, eEF1A and the ribosome both undergo large-scale conformational changes, mammalian ribosomes undergo a process of subunit ‘rolling’ of which observation is lacking in bacteria^15,18^. The role and timing of these conformational events in human, and how eukaryote-specific features of decoding contribute to fidelity, are presently unclear.

Here we reconstitute human translation reactions in vitro^19^ to examine the molecular basis of decoding at a high temporal and spatial resolution by combining multiperspective single-molecule fluorescence resonance energy transfer (smFRET) imaging and cryogenic electron microscopy (cryo-EM). Together, these methods reveal that decoding in human is ten times slower than in bacteria and is rate limited by conformational events during proofreading selection. Although the decoding reaction is globally conserved, fidelity in human is governed by rapid and reversible SSU domain closure and rolling processes, structurally distinct from those evidenced in bacteria. Moreover, eukaryote-specific interactions of eEF1A have critical roles in guiding cognate aa-tRNA to the physically separated decoding, GTPase-activating and peptidyltransferase centres, rationalizing more accurate decoding. These findings shed light on the molecular mechanisms by which clinically relevant small molecules target human protein synthesis and reveal how decoding may be subjected to cellular regulation^20,21^.

## Real-time imaging of human mRNA decoding

**Extended Data Fig. 1:**
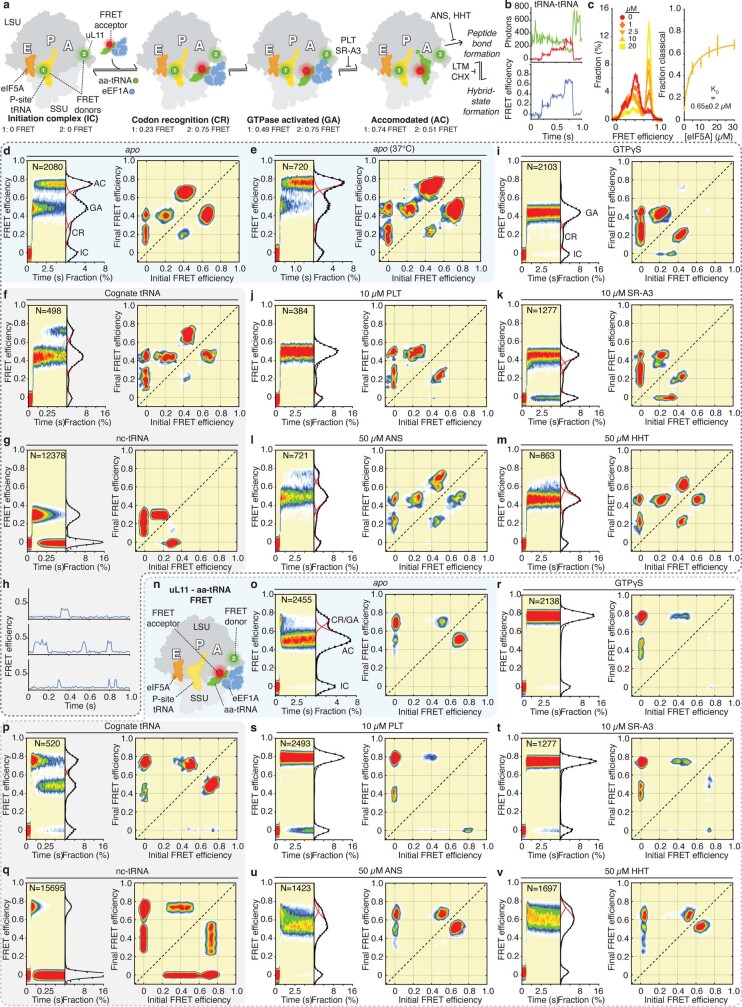
Overview of smFRET data showing stalling of decoding by small-molecule interventions and near-cognate aa-tRNA from two structural perspectives. **a**, Schematic of the decoding reaction on human ribosomes as observed by smFRET. A reaction coordinate of four relatively long-lived ribosome states is inferred, (1) IC with an empty A site, (2) CR with ternary complex bound to the SSU A site but not the LSU, (3) GA with ternary complex bound to the SSU and docked at the LSU GTPase activating centre (GAC) and (4) AC without eEF1A and with A-site tRNA fully accommodated into the peptidyl transferase centre (PTC). Donor and acceptor fluorophore locations and FRET efficiencies for each state in the two FRET perspectives employed are indicated. Locations along the reaction coordinate inhibited by the drugs plitidepsin (PLT), SR-A3, anisomycin (ANS), homoharringtonine (HHT), cycloheximide (CHX) and lactimidomycin (LTM) are indicated. **b**, Example fluorescence (*top*) and FRET efficiency time traces (*bottom*) from the perspective of FRET between the two tRNAs at 10 ms time resolution in the absence of any inhibition. **c**, 1D FRET efficiency population histograms of pre-translocation complexes imaged at different concentrations of eIF5A (*left*) and fraction of classical-state (~0.7 FRET efficiency) ribosomes as a function of eIF5A concentration (*right*) estimated by fitting of a sum of gaussian functions to data such as on the left. The solid line represents a fit of an equilibrium binding equation (SI) to the data. Error bars represent SEM from duplicate experiments. **d**, 2D and 1D population FRET histograms (*left*) and transition density plot (*right*) for an uninhibited decoding reaction imaged at a time resolution of 100 ms at 25 °C. **e**, 2D and 1D population FRET histograms (*left*) and transition density plot (*right*) for an uninhibited decoding reaction imaged at a time resolution of 40 ms at 37 °C. On average, (d) the uninhibited reaction proceeds rapidly through the CR and GA states to reach the AC state. However, the transition density plots indicate that reversible transitions between states are common prior to forward progression along the reaction coordinate. **f**, 2D and 1D population FRET histograms (*left*) and transition density plot (*right*) for the cognate tRNA decoding reaction imaged at 10 ms time resolution from the tRNA–tRNA FRET perspective. **g**, 2D and 1D population FRET histograms (*left*) and transition density plot (*right*) for the near-cognate tRNA decoding reaction imaged at 10 ms time resolution from the tRNA–tRNA FRET perspective. **h**, Example traces of near-cognate decoding events imaged at 10 ms time resolution from the tRNA–tRNA structural perspective. **i–m**, 2D and 1D population FRET histograms (*left*) and transition density plots (*right*) for decoding reactions imaged at a time resolution of 100 ms from the tRNA–tRNA FRET perspective with inclusion of the indicated stalling agents. (i) The slowly hydrolysable GTP analogue GTPγS stalls the reaction in the GA state with fluctuations to the CR state. The eEF1A-dissociation inhibitors (j) PLT and (k) SR-A3 likewise stall the reaction predominantly in the GA state with rare excursions to the CR state. The peptidyl transferase inhibitors (l) ANS and (m) HHT stall the reaction in a GA-like state with rare excursions to the CR and AC states. Red and black lines in the 1D FRET histograms indicate fits of gaussian functions to the data. **n**, Schematic of the uL11-tRNA FRET perspective. **o**, 2D and 1D population FRET histograms (*left*) and transition density plots (*right*) for an uninhibited decoding reaction imaged at a time resolution of 100 ms from the uL11-tRNA FRET perspective. On average, (o) the uninhibited reaction proceeds rapidly through the CR/GA states to reach the AC state. However, the transition density plot indicates that reversible transitions between states are common prior to forward progression along the reaction coordinate. **p**, 2D and 1D population FRET histograms (*left*) and transition density plot (*right*) for the cognate tRNA decoding reaction imaged at 10 ms time resolution from the uL11-tRNA FRET perspective. **q**, 2D and 1D population FRET histograms (*left*) and transition density plot (*right*) for the near-cognate tRNA decoding reaction imaged at 10 ms time resolution from the uL11-tRNA FRET perspective. **r–v**, 2D and 1D population FRET histograms (*left*) and transition density plots (*right*) for decoding reactions imaged at a time resolution of 100 ms from the uL11-tRNA FRET perspective with inclusion of the indicated stalling agents. (r) The slowly hydrolysable GTP analogue GTPγS stalls the reaction in the GA state with fluctuations to the CR state. The eEF1A-dissociation inhibitors (s) PLT and (t) SR-A3 likewise stall the reaction predominantly in the GA state with rare excursions to the CR state. The peptidyl transferase inhibitors (u) ANS and (v) HHT stall the reaction in a GA-like state with rare excursions to the CR and AC states.

To investigate the kinetics and structural dynamics of human decoding, we used multiperspective smFRET imaging (Methods and Extended Data Fig. 1a,b). Building on previous investigations^8,19^, translation was initiated non-enzymatically on synthetic mRNA using purified human ribosomal subunits and initiator Met-tRNA^fMet^. We specifically examined the first elongation cycle in which eukaryotic initiation factor 5A1 (eIF5A) occupies the ribosomal exit site (E site)^22,23^ (Extended Data Fig. 1a,c).

**Fig. 1:**
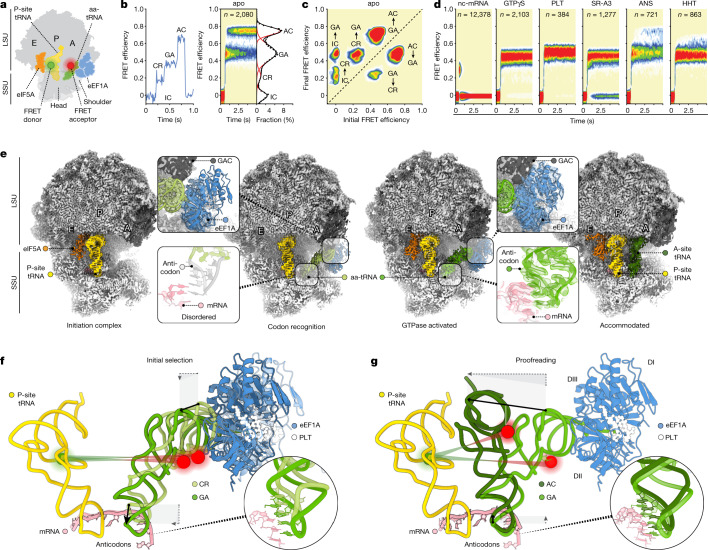
smFRET and cryo-EM investigations of structural dynamics during mRNA decoding. **a**, Schematic of FRET donor (P-site tRNA) and acceptor (aa-tRNA) fluorophores. **b**, Example smFRET data (10-ms time resolution) of a decoding reaction from the perspective shown in **a** showing progression from IC to CR and GA to AC for a single trace (left), and a population histogram of *n* traces (right). **c**, Transition density plot showing the FRET efficiency before and after each transition detected in this population of traces using hidden Markov model (HMM) idealization. **d**, Population histograms as in **b** of decoding reactions in the presence of an mRNA displaying a near-cognate (nc) A-site codon, GTPγS, PLT (10 µM), SR-A3 (10 µM), ANS (50 µM) or HHT (50 µM), showing stalling or rejection along the reaction coordinate. **e**, Overview of four cryo-EM reconstructions along the decoding reaction coordinate, filtered by local resolution and contoured at 3*σ*. Insets: details of eEF1A interacting with the GAC (top; 3*σ*) and the aa-tRNA (Phe-tRNA^Phe^) ASL (bottom; 4*σ*) in the CR-to-GA transition. **f**,**g**, tRNA motions in the transition between the CR and GA complexes (**f**) and the GA and AC complexes (**g**) showing the positions of the FRET label attachment points and the distance between them, coloured as in **a**.

We first stop-flow-delivered acceptor-labelled (LD655) Phe-tRNA^Phe^ in ternary complex with eEF1A and GTP to initiation complexes (ICs) with donor-labelled (Cy3) P-site Met-tRNA^fMet^ (Fig. 1a)^8^. Consistent with previous studies^8,12,19^, decoding stochastically and reversibly progressed through three discernible states with distinct FRET efficiencies (FRET = 0.23 ± 0.09, 0.49 ± 0.13 and 0.74 ± 0.06; Fig. 1b,c). These findings support the existence of at least four evolutionarily conserved and structurally distinct ribosome conformations during decoding, including two ternary-complex-bound intermediates differentiated by the distance between the aa-tRNA and the P-site tRNA^8,12,19^ (Extended Data Fig. 1a).

Ternary complex binding to the human ribosome occurred with an apparent bimolecular rate of 70 ± 6 µM^−1^ s^−1^. Formation of the final high-FRET, fully accommodated PRE complex^19^ occurred with a catalytic efficiency of 43 ± 3 µM^−1^ s^−1^ (Supplementary Table 1). Thus, although the decoding mechanism is globally conserved, the apparent rate of ternary complex binding was about twofold lower compared with bacteria^8^. Moreover, most cognate aa-tRNA binding events were productive, whereas non-productive dissociation is the predominant pathway in bacteria^8^. Notably, intermediate-to-high-FRET transitions immediately before PRE complex formation were around tenfold slower compared with in bacteria^8,11^ (1.7 ± 0.2 s^−1^ versus about 30 s^−1^ at 25 °C, 12.8 ± 2.7 s^−1^ versus around 130 s^−1^ at 37 °C; Extended Data Fig. 1d,e and Supplementary Table 1). These findings suggest physical distinctions in the rate-limiting conformational events underpinning human decoding^8,11,12^.

## Proofreading is rate limiting in human

To establish the origin of slower decoding in human, we first confirmed biochemical assignment of the low-FRET and intermediate-FRET states on the decoding reaction coordinate (Fig. 1d and Extended Data Fig. 1d–h). Decoding reactions performed with ribosomes that were programmed with a near-cognate mRNA codon (UCU instead of UUC) in the A site featured predominantly short-lived, low-FRET events. Low-FRET states therefore probably represent early codon-recognition (CR) states during initial selection^8,12^. Decoding was stalled in intermediate-FRET states by the slowly hydrolysing GTP analogue GTPγS, as expected for GTPase-activated (GA) states^19,24^. Nearly identical inhibitory effects were observed by addition of the cyclic peptides plitidepsin (PLT) and SR-A3, whose related compounds didemnin B and ternatin-4 trap ternary complex on the ribosome immediately after eEF1A-catalysed GTP hydrolysis^24–26^. The PTC inhibitors anisomycin (ANS) and homoharringtonine (HHT)^27,28^ also efficiently slowed intermediate-to-high-FRET progression, consistent with unstable aa-tRNA accommodation^29^. These results are consistent with reduced decoding speed in human, principally deriving from slower proofreading selection after GTP hydrolysis.

In the presence of GTPγS, cognate decoding reactions exhibited reversible excursions between the CR and GA states (Extended Data Fig. 1i). As observed in uninhibited reactions, CR-state lifetimes of cognate decoding reactions in the presence of GTPγS were commensurate with the experimental time resolution (around 10 ± 5 ms; Methods), whereas GA-state lifetimes were relatively long (620 ± 40 ms; Supplementary Table 1). By contrast, near-cognate decoding events exhibited a broad range of low-FRET efficiencies spanning between CR- and GA-state FRET values (Extended Data Fig. 1g,h). These findings are consistent with rapid unsuccessful attempts by near-cognate aa-tRNA to reach a stable GA state. Sharp reductions in the GA-state lifetime then ultimately result in non-productive near-cognate aa-tRNA dissociation.

We corroborated these CR- and GA-state assignments by imaging decoding reactions from a second structural perspective in which the donor fluorophore (LD555) was enzymatically linked to ribosomal protein uL11 within the GAC (Methods and Extended Data Fig. 1n). As in bacteria^8^, this perspective revealed that PRE-complex formation occurs through progression from high-FRET (0.75 ± 0.07) to intermediate-FRET (0.51 ± 0.12) states (Extended Data Fig. 1o). Passage through high-FRET states was efficiently blocked by GTPγS, PLT and SR-A3, and slowed by ANS and HHT (Extended Data Fig. 1r–t). Near-cognate aa-tRNA decoding was characterized by transient excursions to high-FRET (Extended Data Fig. 1p,q). We conclude that the CR and GA states exhibit indistinguishable FRET values when imaged from this structural perspective, consistent with uL11 and the GAC accompanying ternary complex movements during initial selection^9^.

Both smFRET structural perspectives revealed a multi-step decoding reaction coordinate defined by rapid and reversible movements of aa-tRNA between physically distinct positions within the A site. As in bacteria^8^, the rates and efficiencies of these structural transitions strongly depend on proper mRNA codon–tRNA anticodon (codon–anticodon) pairing. Cognate aa-tRNAs rapidly and productively navigate initial selection facilitated by GAC motions to trigger GTP hydrolysis by eEF1A. Conformational processes within the ternary complex and the ribosome during proofreading selection occur more slowly and are rate limiting to PRE-complex formation.

## Human mRNA decoding captured by cryo-EM

To establish the molecular recognition events underpinning decoding kinetics and fidelity in human, we plunge-froze pre-steady-state decoding reactions for structure determination by cryo-EM (Methods). To capture rapidly transited intermediates, we included GTPγS, PLT and ANS, while hybrid-state PRE complex formation was suppressed by inclusion of the LSU E-site-binding drug, lactimidomycin (LTM).

**Extended Data Fig. 2:**
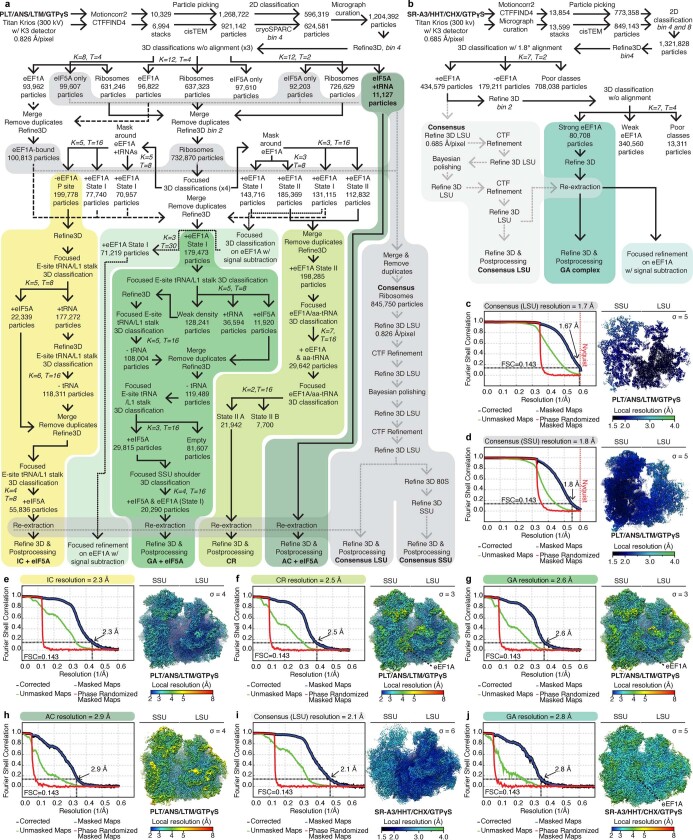
Cryo-EM data processing and refinement of the ribosome structures. **a**, **b**, Flowcharts of cryo-EM data processing of the ribosome structures stalled with (a) plitidepsin, anisomycin, lactimidomycin and GTPγS (PLT, ANS, LTM and GTPγS) and (b) SR-A3, homoharringtonine, cycloheximide and GTPγS (SR-A3, HHT, CHX and GTPγS). For 3D classifications, the number of classes (K) and the regularization parameter (T) are indicted. For focused 3D classification and refinements, regions contained within the soft mask are indicated. To generate high-resolution consensus maps, the particles circled in light grey line were merged, pooled, filtered for duplicates and refined with the full pixel sizes. Focused refinements with signal subtraction and 3D classifications with signal subtraction were performed with ‘shiny’ particles reextracted from refined consensus LSU metadata. All processing was conducted in RELION 3.1^68^, unless otherwise noted. Processing of consensus maps and final refinements/postprocessing of all maps was conducted in RELION 4.0^70^. **c**–**j**, Cryo-EM maps filtered and coloured by local resolution (*left*) and Fourier shell correlation (FSC) curves (*right*) obtained by masking the two half maps and calculating the cross-correlation between the masked volumes^75^ in RELION 4.0 for (c) consensus LSU, (d) consensus SSU, (e) initiation (IC), (f) CR, (g) GA and (h) AC complexes stalled with PLT, ANS, LTM and GTPγS and (i) consensus LSU and GA complexes stalled by SR-A3, HHT, CHX and GTPγS. Resolution was estimated using the 0.143 cutoff criterion (black dotted line). Cross-validation was used to optimize the weight on the experimental density in REFMAC to prevent overfitting^73^. Cryo-EM density contour levels are indicated in σ units. Refinement procedures are described in **Methods**. For more details on cryo-EM processing, see also Extended Data Table 1.

**Extended Data Fig. 3:**
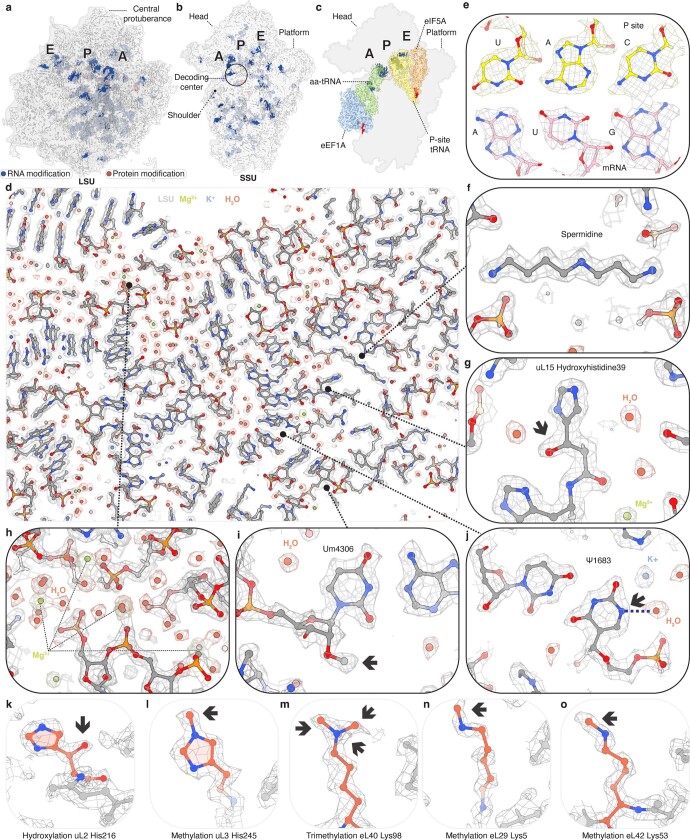
High-resolution structural features observed in the human ribosome consensus reconstruction. **a**–**c**, Cryo-EM density of complexes stalled with plitidepsin, anisomycin, lactimidomycin and GTPγS (PLT, ANS, LTM and GTPγS) highlighting the locations of visualized post-transcriptional (dark blue) and post-translational (dark red) modifications on (a) the LSU and (b) the SSU from the high-resolution consensus reconstruction and (c) aa-tRNA (Phe-tRNA^Phe^), P-site tRNA (Met-tRNA^fMet^), eEF1A and eIF5A from the GTPase activated reconstruction. **d**–**n**, High-resolution structural features visible within the consensus cryo-EM map, including (d) the centre of the LSU core, (e) the start codon–anticodon nucleotides in the P site (f) polyamines, (g, k) hydroxylhistidines, (h) fully hydrated Mg^2+^ ions (lime green, *left*) and partially hydrated and fully coordinated and Mg^2+^ ions (*top*, *middle* and *right*), (i) 2′O-Me uridine (Um), (j) pseudouridine (Ψ), (l) methyl-histidine, (m) trimethyl-lysine and (n, o) methyl-lysine. Protein and nucleic acid modifications are indicated with black arrows. Cryo-EM density is from post-processed high-resolution consensus LSU map and is contoured at 3 σ for panel m and 5 σ for all remaining panels. See also Methods, Extended Data Fig. 2 and Extended Data Table 1.

Classification of the cryo-EM dataset (Extended Data Fig. 2) revealed a population of particles with strong density for P-site Met-tRNA^fMet^. Independent, focused refinement on the LSU and SSU yielded reconstructions that resolved to 1.67 Å and 1.89 Å, respectively (Extended Data Table 1). We used these data to aid atomic model building, including P-site tRNA pairing with the start codon, ions, water molecules, polyamines and 218 out of 230 biochemically verified post-transcriptional modifications, including 104 pseudouridines^30^ (Extended Data Fig. 3). Further classification resulted in four reconstructions that refined to about 2.3–2.9 Å, exhibiting compositional and conformational properties consistent with those predicted by smFRET (Extended Data Fig. 2, Extended Data Table 1 and Supplementary Videos 1 and 2). These complexes reflect the IC state before ternary complex binding, the transient ternary complex-bound CR state, the GA state immediately before GTP hydrolysis and the fully accommodated, classical PRE complex (hereafter, AC) (Fig. 1e).

## P-site tRNA stabilization in human

**Extended Data Fig. 4:**
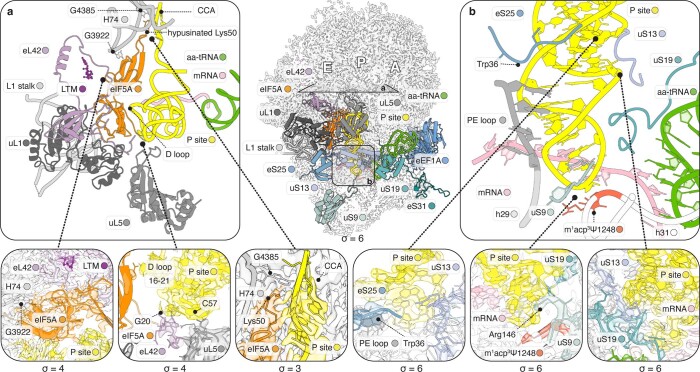
Interactions between eIF5A, P-site tRNA and the human ribosome. **a**, Overview of the E site from the indicated orientation (*centre*), showing interactions between uL1, uL5, eL42, eIF5A, the L1 stalk rRNA, H74 and P-site tRNA. Lactimidomycin (LTM) shown in ball-and-stick representation. Insets running left to right show zoom-ins of the position of eIF5A relative to LTM, H74 base G3922, and eL42 (*left*), the interactions between P-site tRNA base pair G20-C57 and uL5/eL42 and P-site tRNA D-loop residues 16-21 interaction with eIF5A (*middle*) and the CCA-end of P-site tRNA (*right*). **b**, Overview of the P-site tRNA acceptor stem from the indicated direction showing interactions with eS25 (N terminus), uS13 (C terminus), uS19 (C terminus), uS9 (C terminus) and SSU bases of h31 (m^1^acp^3^Ψ1248, red) and h29 (1639-1642, PE loop). Insets running left to right show zoom-ins of interactions between the P-site tRNA and: eS25, uS13 and the PE loop (*left*); mRNA, uS9 and m^1^acp^3^Ψ1248 (*middle*); and uS19 and uS13 (*right*). Cryo-EM density and atomic model are from the GTPase activated complex. All cryo-EM density is filtered by local resolution and is contoured in units of σ as indicated.

In the IC structure, classical ribosome conformations were enforced by both eIF5A and ribosome contacts with initiator tRNA in the P site (Extended Data Fig. 4). The SSU was therefore unrotated and unrolled relative to the LSU. The SSU shoulder domain occupied an open position^10^, leaving the E site relatively compacted and the A site relatively open^18^. The central domain of uL1 collapsed over the eIF5A-binding pocket towards the SSU to contact the C terminus of eIF5A, the eukaryote-specific ribosomal protein eL42 and uL5 at the LSU central protuberance, closing the E site^23,31^ (Extended Data Fig. 4a). The eIF5A N terminus wedged against the P-site tRNA D loop, eL42 and LSU rRNA helix 74 (H74). P-site tRNA was further secured by interactions with eL42 and uL5 through the highly conserved base pair at the apex of the tRNA elbow (G20–C57 in tRNA^fMet^)^32^. Consistent with stabilizing P-site tRNA for efficient peptide bond formation^22^, the hypusinated Lys50 of eIF5A buttressed 3′-CCA interactions with the PTC.

As observed in other species^33^, the P-site tRNA ASL engaged uS9 and universally conserved SSU head domain elements, while its codon–anticodon pair contacted the post-transcriptionally modified SSU rRNA nucleotide m^1^acp^3^Ψ1248 (m^2^G966 in *Escherichia coli*) (Extended Data Fig. 4b). In contrast to in bacteria, terminal elements of four additional ribosomal proteins of the SSU head domain extended into the A site (eS31) and P site (uS13, uS19 and eS25), the latter of which interacted directly with P-site tRNA. This complement of stabilizing interactions secured P-site tRNA in all four reconstructions, rendering it stationary throughout the decoding process. FRET changes accompanying decoding therefore principally report on ternary complex and aa-tRNA movements within the A site.

## Distinct trajectory of aa-tRNA motion

**Extended Data Fig. 5:**
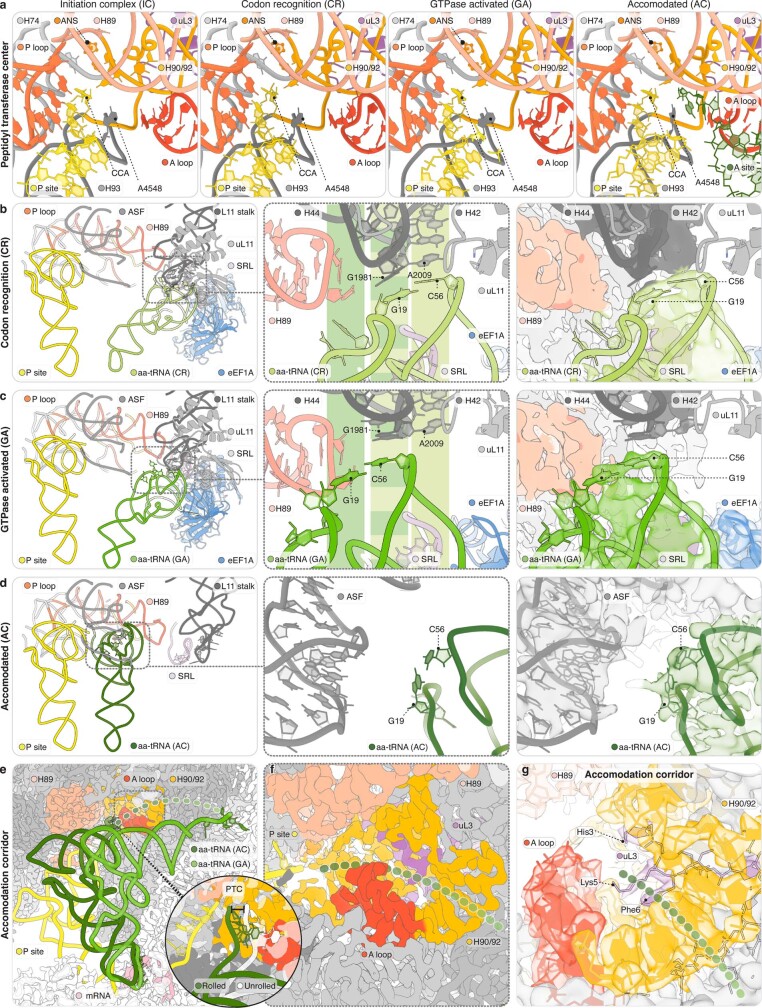
Remodelling of interactions between the aa-tRNA and the ribosome during mRNA decoding. **a**, Interactions between the tRNA CCA-ends and the peptidyl transferase centre (PTC). **b**–**d**, Overview of aa-tRNA interactions with the L11 stalk (H42 and H44), H89 and the LSU A-site finger (ASF) in (b) the CR, (c) GA and (d) AC complexes stalled with plitidepsin, anisomycin, lactimidomycin and GTPγS. The relative position of aa-tRNA and P-site tRNA and the ASF, H89 and the GTPase activating centre (GAC, *left*). A zoom in on the region within the dotted line is provided with (*right*) and without (*middle*) experimental density, showing the interaction with aa-tRNA (Phe-tRNA^Phe^) bases G19 and C56. The vertical green fields show the overall position of the tRNA bases G19 and C56 relative to the LSU. These show the stacking between G19 and C56 of the aa-tRNA elbow with G1981 of H44 and A2009 of H42, respectively, in the CR complex. In the GA complex the aa-tRNA has moved past H89 further towards the P site and the SSU, shifting the stacking interaction between the elbow and H42 and H44 such that C56 of the elbow now stacks on G1981 of H44 while G19 no longer makes any stacking interaction. In the AC complex, G19 and C56 interact weakly with the ASF. **e**, View of aa-tRNA accommodation showing the spatial relationship between the aa-tRNA, the P-site tRNA and the accommodation corridor. The inset shows the movement of the aa-tRNA CCA end due to subunit rolling, the white tRNA is a model of an accommodated tRNA with its anticodon stem loop (ASL) aligned onto that of an aa-tRNA on an unrolled ribosome in the GA complex. The dashed green line shows the likely path of the aa-tRNA CCA end. **f**, **g**, Zoom-ins of the accommodation corridor corresponding to the dashed rectangle in (e) showing (f) experimental cryo-EM density and (g) atomic model showing the additional crowding due to the eukaryote extension of uL3. All cryo-EM density is filtered by local resolution and is contoured at 3 σ.

As evidenced in organisms ranging from bacteria to rabbit^8,12–14,18,34^, the ternary complex moved relative to P-site tRNA and the ribosome core during both initial selection (CR to GA) and proofreading selection (GA to AC) (Fig. 1e). In the CR complex, the ternary complex was positioned to orient the aa-tRNA ASL towards the SSU decoding centre. Although close enough for contact, both the mRNA codon and aa-tRNA anticodon were unstructured and the G domain of eEF1A was physically separated from the catalytic SRL (Fig. 1e (insets)). By contrast, aa-tRNA in the GA complex exhibited a fully structured codon–anticodon pair^24^, while the G domain of eEF1A packed against the SRL^9,24^. In the AC structure, eEF1A was absent and aa-tRNA was classically positioned within the PTC, in which its 3′-CCA end engaged the highly conserved A-loop^33^ (Fig. 1e and Extended Data Fig. 5a). Consistent with ANS sterically inhibiting peptide-bond formation^27^, the nascent chain was not visible.

**Extended Data Fig. 6:**
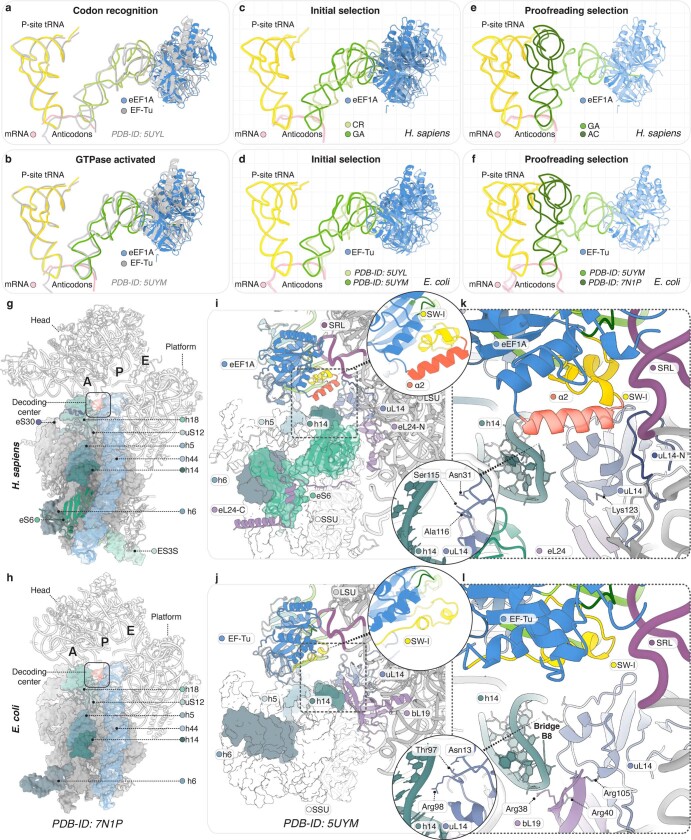
tRNA positions and ternary complex interactions in decoding intermediate complexes in human and bacteria. **a**, **b**, Superposition of early (CR) (**a**) and late (GA) (**b**) ternary complex-bound decoding intermediates from human (coloured) and bacteria from (grey; ‘Structure II’, PDB-ID: 5UYL^13^; ‘Structure III’, PDB-ID: 5UYM^13^). **c**, **d**, tRNA motions in the transition between the CR and GA complexes (**c**) and the GA and AC complexes (**d**) in human. **e**, **f**, tRNA motions in the transition between ‘Structure II’ in reference^14^ and ‘Structure III’ in reference^14^ (**e**) and ‘Structure III’ in reference^14^ and PRE-C in bacteria^38^ (**f**). Alignment on the P-site tRNA. **g**, **h**, Overview of the SSU in human (AC complex) (**g**) and bacteria (PRE-C, PDB-ID: 7N1P^38^) (**h**) showing the size of the mobile shoulder domain (surface representation). The decoding centre is shown in red. **i**, **j**, Overview of the absence (human, GAcomplex) (i) and presence (bacteria, ‘Structure III’, PDB-ID: 5UYM^13^) (**j**) of intersubunit bridge B8 between h14, uL14 and bL19 (bacteria only). The shoulder domain is shown in surface representation. *Insets*, comparison of switch-I (SW-I) in eEF1A (*left*) and EF-Tu (*right*). **k**, **l**, Close-up view of bridge B8, same structures as in (i, j) for human (**k**) and bacteria (**l**). Alignment on the LSU core.

As inferred from smFRET imaging, initial selection shifted the aa-tRNA elbow domain by about 7 Å into the intersubunit space towards the P-site tRNA through tRNA sequence-independent contacts with the GAC (Fig. 1f and Extended Data Fig. 5b,c). During proofreading selection, the aa-tRNA elbow shifted by another approximately 26 Å towards the P site, due in part to relaxation of aa-tRNA bending present in the GA structure, which may ‘spring load’ the accommodation step^35^ (Fig. 1g and Extended Data Fig. 5c,d). Accompanying movements towards the P site, aa-tRNA followed an additional vector of motion perpendicular to the intersubunit space during decoding, moving towards the SSU during initial selection and towards the LSU during proofreading selection (Fig. 1f,g). This additional vector of motion, which has not been observed in bacteria^13,14^ (Extended Data Fig. 6a–f) and was not directly revealed by the present smFRET structural perspectives, may be specific to decoding in eukaryotes and perhaps mammals explicitly.

## Initial selection is distinct in human

**Fig. 2:**
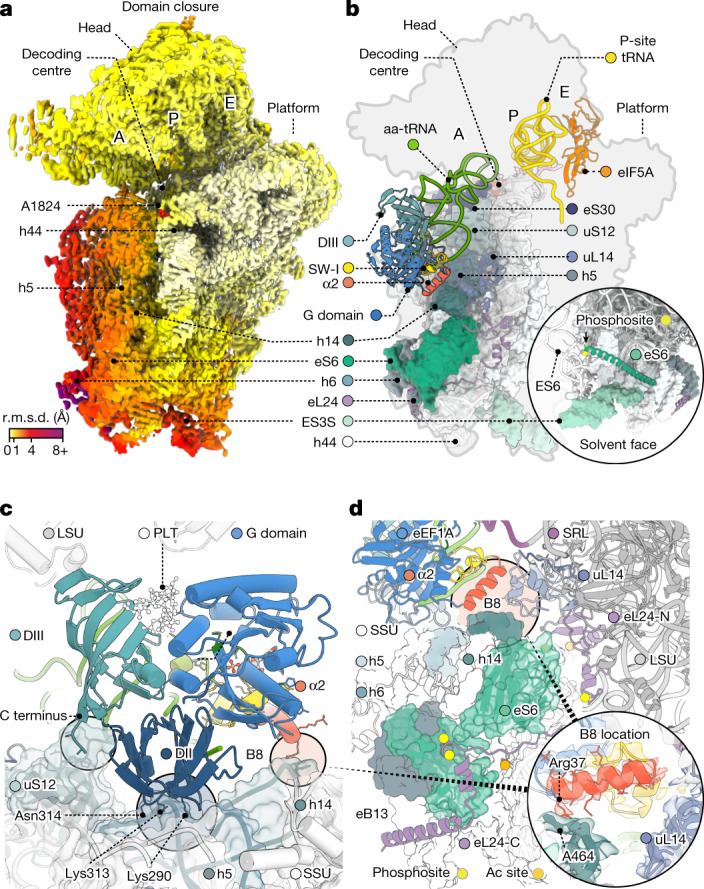
Domain closure and initial ternary complex binding. **a**, Cryo-EM density of the SSU from the GA complex coloured by backbone root-mean-squared deviation (r.m.s.d.) compared with the CR complex, contoured at 3*σ*. **b**, Overview of the SSU from the GA complex, showing the positions of the ternary complex, P-site tRNA and eIF5A and illustrating the size of the mobile shoulder domain (surface representation). Inset: solvent-exposed post-translationally modified C-terminal helix of eS6; known phosphorylation sites on the structured part of the C terminus are indicated in yellow. **c**, Magnification of eEF1A contacts with the SSU (surface representation) in the CR complex. The PLT-binding site is indicated. **d**, Overview of missing intersubunit bridges in the CR complex. Known phosphorylation and acetylation (Ac, orange) locations on eL24 are shown as spheres. SSU is shown in surface representation. Inset: atomic model and cryo-EM density illustrating the separated elements of bridge B8 and the α2 helix of eEF1A in the CR complex. Cryo-EM density is contoured at 3*σ*. Alignment is on the LSU core.

Productive eEF1A engagement of the GAC at the end point of initial selection was accompanied by rotation and compaction of the SSU shoulder domain, relative to the long axis of SSU h44, towards the SSU head domain and the GAC^10,13,14,18,24^ (Fig. 2a). The mobile SSU shoulder domain was considerably larger in human than in bacteria, encompassing around 600 rRNA residues and 7 ribosomal proteins, including both the leading edge of the body domain as well as expansion segment 3S (ES3S; Fig. 2b and Extended Data Fig. 6g–j). Notably, ES3S extends from the solvent surface of the SSU towards the E-site lagging edge by wrapping around the C-terminal tail of ribosomal protein eS6, a target of cancer-relevant intracellular signalling pathways^36^.

In the CR structure, the interactions between the ternary complex and the ribosome were sparse and tRNA-sequence independent, consistent with unstable binding (Fig. 2c). As in bacteria^13,14,18,37^, domain II of eEF1A nested against SSU helix 5 (h5) of the shoulder domain while the C terminus of eEF1A (domain III) engaged in a handshake-like arrangement with the C terminus of uS12. Moreover, the G19–C56 base pair within the aa-tRNA elbow^32^ contacted rRNA elements of the GAC, which structured and moved towards the P site and SSU (Extended Data Fig. 5b). In human, these CR-complex contacts were buttressed by an additional interaction site between h14 of the SSU shoulder domain and the eukaryote-specific α2 helix that extends from the N-terminal portion of switch I of eEF1A (Fig. 2c,d). This contact cannot occur in bacteria as the homologous translation factor EF-Tu lacks an α2 helix equivalent (Extended Data Fig. 6i–l).

Intersubunit bridge 8 (B8), formed principally between h14 and uL14, remained broken throughout initial selection as a consequence of the ‘unrolled’ ribosome conformation of the IC, CR and GA structures (Fig. 2d). Adding to these distinctions, bacterial-specific ribosomal protein bL19—a component of B8 in bacteria—is replaced by eukaryote-specific ribosomal protein eL24 in human (Extended Data Fig. 6i–l). Although eL24 bears partial structural homology to bL19, it lacks N-terminal residues that are critical to mediating h14 contact. C-terminal elements of eL24 instead principally contributed to eB13, contacting h6 adjacent to B8, while the approximately 60 amino acid extension of eL24 engaged more distal elements of h6 near ribosomal protein eS6 (Fig. 2d). The absence of B8 during initial selection in human implies that the SSU shoulder domain probably exhibits increased conformational degrees of freedom and that the C-terminal extension of eL24 may serve to tether the SSU shoulder domain relative to the LSU.

## Human decoding centre distinctions

**Fig. 3:**
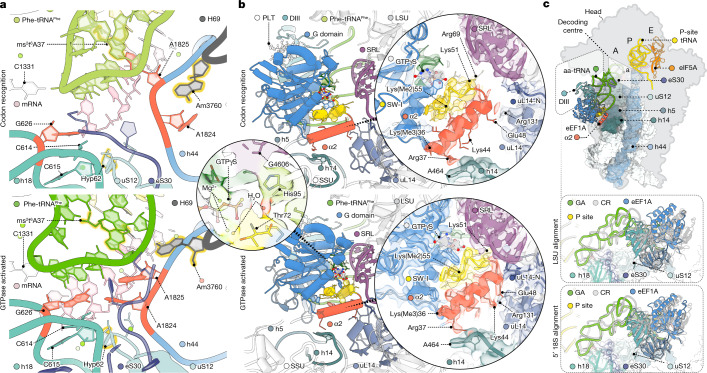
Structural remodelling during initial selection. **a**, Remodelling of the decoding centre to recognize the codon–anticodon helix in the CR (top) to GA (bottom) transition, highlighting the monitoring bases (red) and post-transcriptionally and post-translationally modified residues (yellow outline). **b**, Ternary complex contacts at the subunit interface near bridge B8 in the CR (top) and GA (bottom) complexes. Inset (left): coordination of a catalytic water in the eEF1A G domain in the GA complex; eEF1A-focused refinement. Insets (right): formation of the temporary bridge B8 through the α2 helix of eEF1A. **c**, Overlay of CR (grey) and GA (coloured) complexes showing SSU domain closure and ternary complex movements (top), combined movement of the SSU shoulder and ternary complex (middle; LSU alignment), and ternary complex movements in addition to those induced by SSU shoulder domain closure (bottom; SSU-shoulder alignment). Cryo-EM density is contoured at 3*σ*. Alignment is on the LSU core, unless otherwise noted.

**Extended Data Fig. 7:**
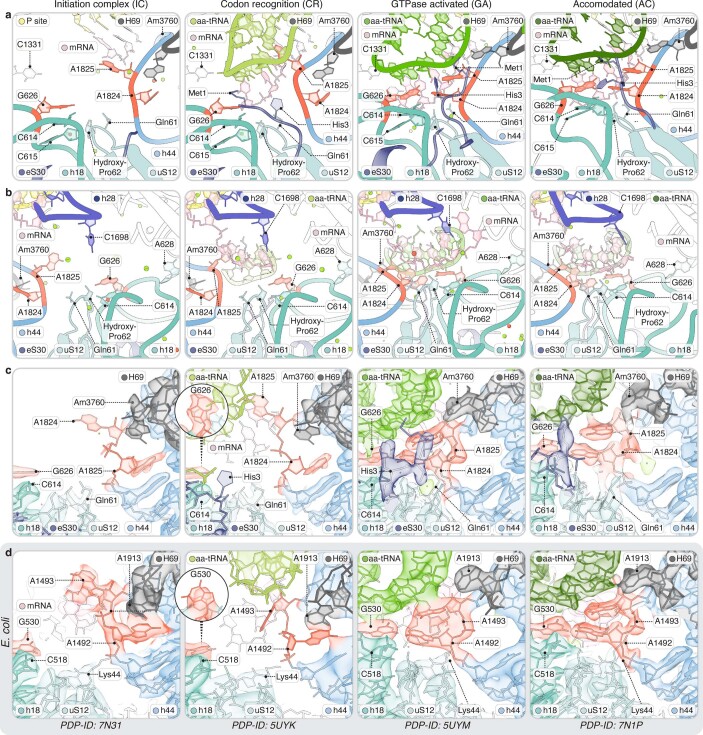
Changes in key decoding centre interactions along the decoding reaction coordinate. **a**–**c**, Views of the decoding centre of IC, CR, GA and AC reconstructions, from left to right. Overview of aa-tRNA (Phe-tRNA^Phe^) in the decoding centre as seen from the leading edge of the SSU (a). The monitoring bases (red) G626, A1824 and A1825 (530, 1492 and 1493 in *E. coli*, respectively) are disengaged in the IC and CR complexes. A1824 resides inside h44, hydrogen bonding between the amino group of Am3760 (A1913 in *E. coli*) of H69 and N3 of A1824 replaces the stacking interaction between these two bases observed in bacteria where Am3760 resides inside h44. A1825 is disordered rather than, as observed in bacteria, flipped out close to its ‘monitoring’ position, possibly due to the empty space available to it inside h44 due to the distal position of Am3760 in human compared to bacteria. G626 is in an anti-conformation, rather than in a syn conformation as observed in bacteria, it is stacked with C614 and positioned away from the decoding centre. Ribosomal proteins uS12 and eS30 are disengaged. In the GA and AC complexes the decoding centre is fully structured around the codon–anticodon minihelix and the monitoring bases occupy positions like those observed in bacteria. A1824 and A1825 reside outside h44 forming A-minor interactions with the codon–anticodon pair. Am3760 has moved away from its CR position and is hydrogen bonded to the aa-tRNA base 3′ of the anticodon, as in bacteria. Domain closure has brought G626, C614 and uS12 roughly 3 Å further into the decoding centre. G626 now hydrogen bonds with A1824 and the first and second bases of the anticodon. C614 coordinates a Mg^2+^ molecule with the third base of the mRNA codon and uS12 hydroxy-pro62. Additionally, uS12 Gln61 forms hydrogen bonds with the second base of the mRNA codon and A1824. C1331 (C1054 in *E. coli*) forms a Pi-stacking interaction with the third base of the anticodon while C1698 (C1397 in *E. coli*) intercalates into the mRNA one base 3′ of the codon. The N-terminal tail of eS30 has become structured and Met1 hydrogen bonds with A1825 while His3 hydrogen bonds to the aa-tRNA residue 3′ of the anticodon and forms a salt bridge to C615. View of the same process from the SSU side highlighting intercalation of C1698 into the mRNA (**b**). Close-up view of the same sequence of events focused on the h44 side of the decoding centre shown with cryo-EM density (**c**). Density for A1824 is strong in all four reconstructions, indicating stable localization and a switch-like behaviour while density for A1825 is absent in IC, weak in CR and strong in GA and AC, indicating a stepwise transition from disordered to ordered positioning of this base. Strong density places G626 in an anti-conformation in all four reconstructions (*inset*), unlike the syn to anti flip observed in bacteria in the CR to GA transition, due to the stacking between G626, C614 and G625 these bases appear to move as a rigid body with the rest of the shoulder domain. **d**, Equivalent cryo-EM structures of decoding intermediates from *E. coli* from the same view as (c). From left to right, POST complex (PDB-ID: 7N31^38^), ‘Structure I’ (PDB-ID: 5UYK^13^), ‘Structure III’ (PDB-ID: 5UYM^13^), PRE-C complex (PDB-ID: 7N1P^38^). All cryo-EM density is filtered by local resolution and is contoured at 5 σ.

SSU domain closure in the CR-to-GA transition locally remodelled the decoding centre to structure and enclose the codon–anticodon pair, engaging the universally conserved SSU rRNA ‘monitoring’ bases G626, A1824 and A1825 (G530, A1492 and A1493 in *E. coli*) (Fig. 3a and Extended Data Fig. 7). Compared with analogous processes in bacteria^10,13,14,38^, we observed differences in the ordering of A1824 and A1825 in the IC complex and the orientation of G626 in the CR complex, suggesting an altered activation barrier for CR.

Consistent with complete codon–anticodon recognition in the GA complex, the neighbouring A628 base (G532 in *E. coli*) within the SSU shoulder domain moved towards the SSU head domain to close the mRNA entrance channel^38^ (Fig. 3a and Extended Data Fig. 7). Codon–anticodon recognition was further secured by stacking of the aa-tRNA anticodon against C1331 (C1054 in *E. coli*) of the SSU head domain and intercalation of C1698 (C1397 in *E. coli*) into the mRNA immediately downstream of the A-site codon^39,40^. Solvent exposure of the codon–anticodon pair was reduced by an outer shell of additional contacts, including the N-terminal portion of eukaryote-specific ribosomal protein eS30 and neighbouring rRNA nucleotides^24^. The codon–anticodon pair also engaged elements of the post-transcriptionally modified H69 base Am3760 (A1913 in *E. coli*), which shifted from being solvent exposed in the CR complex to interacting with the post-transcriptionally modified position 37 of aa-tRNA in the GA complex immediately upstream of the anticodon. Immediately downstream of the last mRNA codon base in the A site, conserved loop elements of ribosomal protein uS12, including Ser64 and post-translationally hydroxylated *cis*-Pro62 residues, also reached into the decoding centre to stabilize stacking of SSU shoulder domain base C614 (C518 in *E. coli*) with monitoring base G626 through magnesium-ion coordination^24^. These observations indicate the potential for post-transcriptional and post-translational modifications to influence initial selection.

## GTPase activation is distinct in human

**Extended Data Fig. 8:**
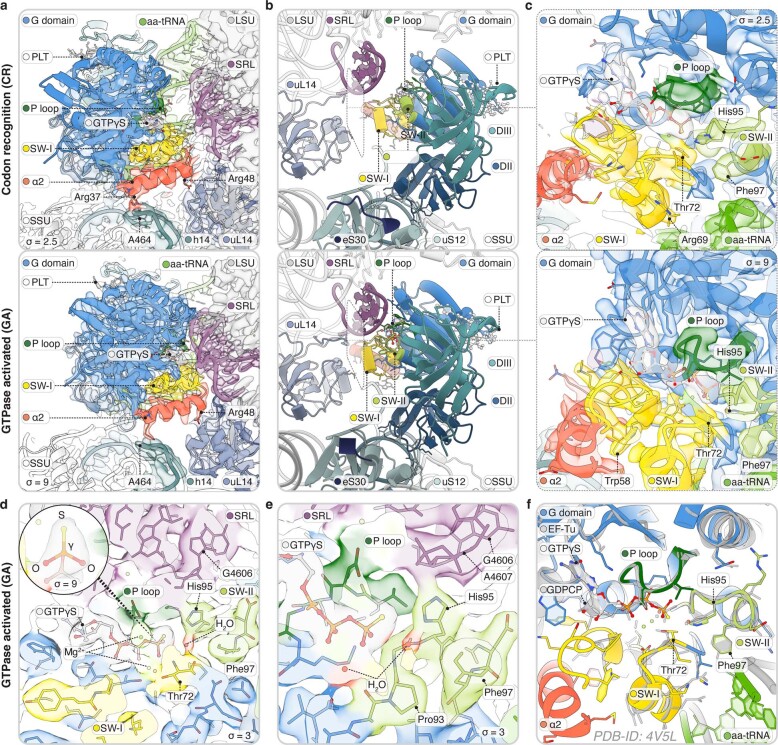
eEF1A interactions with the ribosome in the codon recognition and GTPase activated complexes. **a**, Zoomed-out view of Fig. 3b, showing interactions between eEF1A, the SSU and the LSU in the CR complex (*top*) and the GA complex (*bottom*). In the GA complex the eEF1A G domain packs against the SRL and its α2 expansion segment forms a temporary bridge B8 between h14 and uL14. **b**, View of same process from the head domain of the SSU with aa-tRNA hidden for clarity, showing the interaction between DII of eEF1A with the C-terminus of uS12. **c**, View of the interaction between the eEF1A G domain and the ribosome as seen from the GAC in the CR complex (*top*) and the GA complex (*bottom*). The GTPγS molecule is tightly coordinated in the G-domain by switch I (SW-I), switch II (SW-II), the P loop and other G-domain elements. In the GA complex, the G domain of eEF1A docks onto the GAC, packing the α2 expansion segment tighter against SW-I. The SRL coordinates Arg69 of the SW-I element and the ‘catalytic’ His95 of the SW-II element, respectively, priming eEF1A for GTP hydrolysis. Cryo-EM density from a focused refinement on eEF1A is shown for the GA complex. **d**, **e**, The geometry of the catalytic His95 in the eEF1A G domain in the G interaction between the eEF1A G domain and the ribosome as seen from the GAC in the CR complex (*top*) and the GA complex. Contour levels for cryo-EM density are indicated in σ units.

Ternary complex movement towards the decoding centre in the CR-to-GA transition docked the conserved G domain and catalytic switch-II His95 residue of eEF1A tightly against the SRL (Fig. 3b and Extended Data Fig. 8a,b). Focused refinement with signal subtraction on eEF1A in the GA structure revealed the catalytic geometry of the G domain, including density for a water molecule poised for GTP hydrolysis, and confident modelling of the sulfur position in GTPγS (Fig. 3b and Extended Data Figs. 2 and 8c–e). This G-domain geometry agreed with that of the EF-Tu ternary complex stalled by GDPCP (Extended Data Fig. 8f).

The CR-to-GA transition also remodelled interactions between the G19–C56 base pair of aa-tRNA and the GAC (Extended Data Fig. 5b,c). It also enabled the aa-tRNA elbow to slide past the H89 steric block while remaining engaged with the mobile GAC. This shift in the position of the ternary complex enabled the C-terminal end of the eEF1A α2 helix to directly contact the SRL and to engage and structure the eukaryote-specific C-terminal extension of uL14 (Fig. 3b and Extended Data Fig. 8a,b), bridging the otherwise separated components of B8.

Shoulder domain closure alone was insufficient to explain the full extent of ternary complex movements during initial selection (Fig. 3c). We therefore conclude that GTPase activation in human also requires uncoupled ternary complex movements towards the decoding centre and the SRL to span the increased distance created by the unrolled SSU conformation.

## SSU rolling accompanies proofreading

**Fig. 4:**
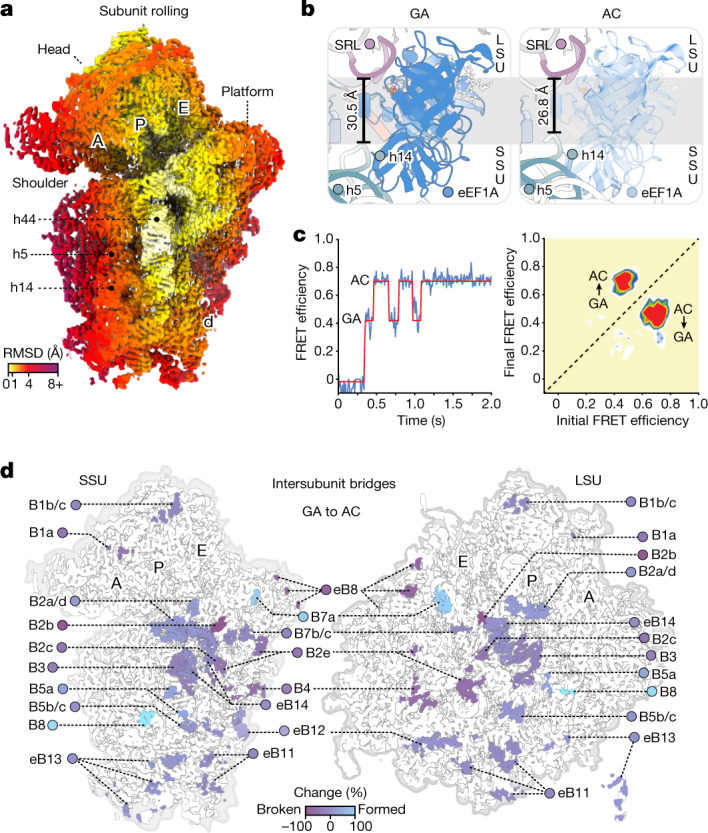
Subunit rolling and tRNA accommodation during proofreading selection. **a**, Cryo-EM density of the SSU of the GA complex coloured by backbone r.m.s.d. compared with the AC complex, contoured at 3*σ*. **b**, Overview of the factor-binding site in the GA (left) and AC (right) complexes, showing closure of the eEF1A-binding site between the SSU and LSU due to SSU rolling. aa-tRNA has been omitted for clarity. The black bar shows the distance between the phosphates of LSU rRNA G4600 and SSU rRNA A464. **c**, Example smFRET trace (blue) and an HMM idealization (red) from the perspective of FRET between the two tRNAs of an uninhibited decoding reaction showing reversible excursions from the GA to the AC state preceding stable AC-state formation (left). Transition density plot showing the FRET efficiency before and after each transition detected in this population of traces by HMM idealization after the first visit to the AC state (right), showing persistent fluctuations back to the GA state. **d**, Intersubunit bridges formed and broken by SSU rolling in the GA-to-AC transition (bottom), contoured at 3*σ*.

Termination of initial selection by eEF1A-catalysed GTP hydrolysis begins the relatively slow stage of proofreading selection. During proofreading selection in human (the GA-to-AC transition) aa-tRNA moved towards the LSU by about 4 Å through SSU rolling (Figs. 1g and 4a). By contrast, proofreading selection in bacteria has not been associated with significant changes in SSU rolling or bridging contacts but has instead been associated with subtle SSU rotation^34^.

SSU rolling also compacted the intersubunit space at the A site, closing the distance between eEF1A’s former contact points on the SSU and LSU (Fig. 4b). These changes require disruption of the B8 contacts mediated by the α2 helix of eEF1A. SSU rolling therefore requires eEF1A to either substantially remodel or dissociate to enable completion of proofreading selection. In this context, drugs targeting eEF1A remodelling^24–26^ efficiently stall eEF1A on the ribosome immediately after GTP hydrolysis^24–26^ (Fig. 1d and Extended Data Fig. 1j,k).

Uninhibited smFRET experiments further revealed direct evidence that this rate-limiting decoding step is associated with rapid, reversible aa-tRNA movements to positions that closely resemble the AC state^12,41^ (Fig. 4c). In bacteria, FRET fluctuations associated with proofreading selection are hypothesized to represent attempts of its CCA-end to navigate the LSU accommodation corridor—a narrow passageway through the A-loop major groove—en route to the PTC^8,42^ (Extended Data Fig. 5e–g). In human, these excursions occurred at rates around fivefold lower compared with in bacteria^8^ (Fig. 4c). We posit that the approximately 5 Å movement of aa-tRNA towards the SSU during initial selection sterically prevents aa-tRNA entry into the LSU accommodation corridor in the absence of SSU rolling (Extended Data Fig. 5e). Furthermore, the accommodation corridor itself is crowded by a eukaryote-specific extension of ribosomal protein uL3^43^ (Extended Data Fig. 5f,g), probably reducing the rate of accommodation.

We infer from these observations that aa-tRNA movements accompanying proofreading selection are associated with eEF1A remodelling-dependent SSU rolling. The higher activation barriers of proofreading selection in human therefore probably arise from the requirement for coincident eEF1A remodelling and SSU rolling to enable aa-tRNA entry into the LSU accommodation corridor. We note that, in this context, eEF1A remodelling during proofreading has been directly observed^15,26^ and that trimethylation of the α2 helix in Ras-driven cancers increases translational output and tumorigenesis^20^.

## A-site/E-site allostery through SSU rolling

**Extended Data Fig. 9:**
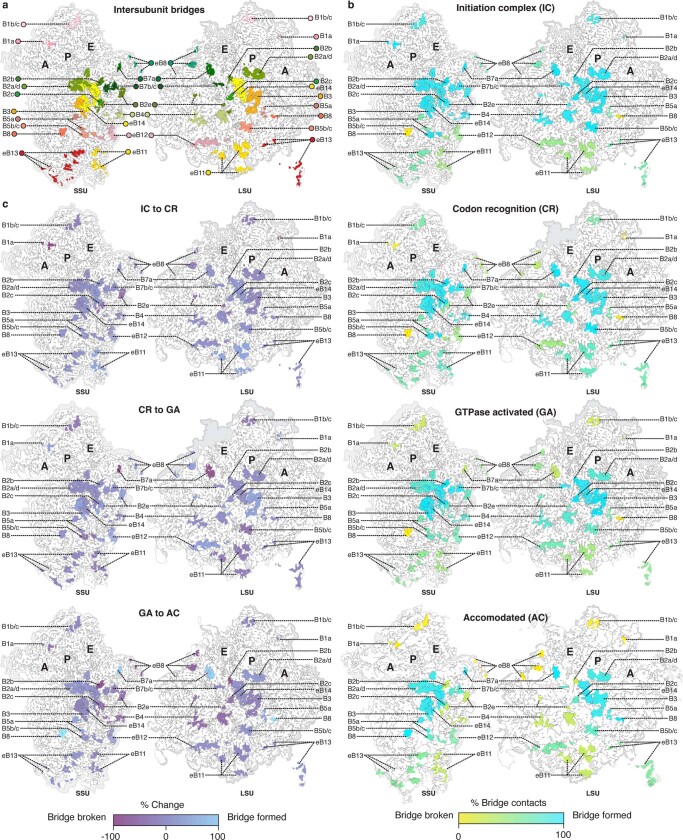
Remodelling of intersubunit bridges during mRNA decoding. **a**, Overview of all intersubunit bridges on the human ribosome. **b**, Overviews of bridges formed (cyan) and not formed (yellow) in the four decoding complexes. **c**, Overview of the changes in bridging interactions during the transitions between the four decoding complexes. All cryo-EM density is filtered by local resolution and is contoured at 3 σ. See Methods for more details.

**Extended Data Fig. 10:**
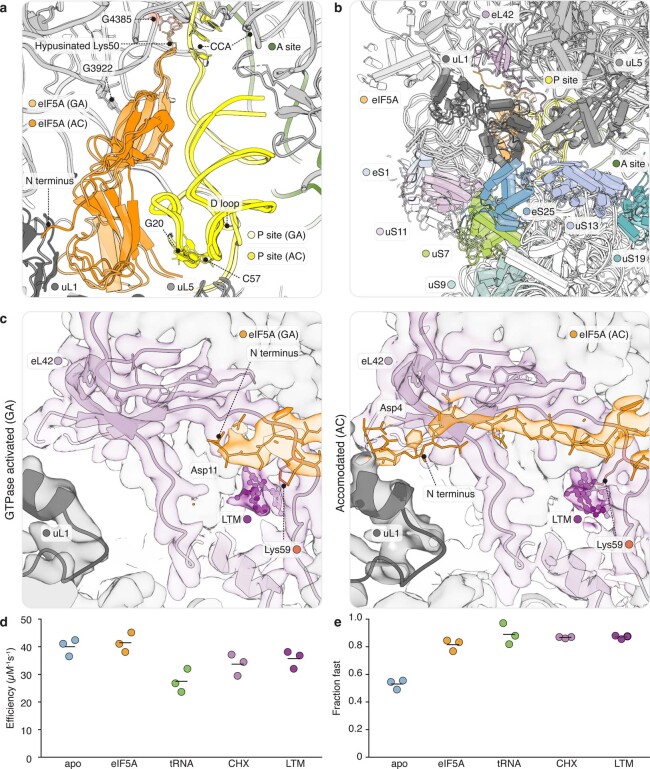
Allosteric interaction between the leading and lagging edges of the ribosome affect mRNA decoding. **a**, Overlay of the interaction between eIF5A and P-site tRNA for the rolled AC complex (solid) and the unrolled GA complex (transparent), showing movements of eIF5A towards the P site and the P-site tRNA towards the E site as a response to SSU rolling. LSU rRNA base G4385 is shown in red. **b**, Overlay of the E site for the rolled AC complex (solid) and the unrolled GA complex (transparent), showing conformational changes in the E site as a consequence of SSU rolling. **c**, Cryo-EM density for the N-terminal tail of eIF5A in the GA (*left*) and AC (*right*) complexes, showing its restructuring as a response to SSU rolling. Cryo-EM density is filtered by local resolution and contoured at 2 σ with a 1 σ gaussian filter. **d**, Catalytic efficiency of decoding on fast ribosomes as a function of the ligand bound in the ribosomal E site, slow ribosomes carried out the decoding reaction with 10-20× lower speed. **e**, Fraction of ribosomes that carried out decoding fast as a function of the ligand bound in the ribosomal E site. Approximately 50% of ribosomes with an empty E site carry out the decoding reaction slowly, whereas with any E site ligand only about 15% do so. This implies that natural as well as small-molecule ligands that bind the E site are able to affect the conformation of the ribosome in a way that accelerates binding of ternary complex to the A site. Each dot represents one experimental replicate, the horizontal bar represents the average.

In bacteria, allosteric communication between the leading and lagging edges of the ribosome (A sites and E sites, respectively) has been a point of debate^44^. In human, SSU rolling during proofreading selection repositions the SSU to remodel intersubunit bridges on both the leading and lagging edges^18^ (Fig. 4d and Extended Data Fig. 9). These changes enable the formation of intersubunit bridges B8 and B7a, and dissociate bridges B2b, B2e and eB8. In so doing, SSU rolling increased the solvent accessibility of the E site, rationalizing stimulation of deacyl-tRNA dissociation^19^, while shifting eIF5A towards the P-site tRNA and the LSU (Extended Data Fig. 10a–c), probably strengthening its interactions with P-site tRNA and eL42. Reciprocally, natural ligands that completely fill the E site (eIF5A and deacyl-tRNA), as well as small molecules that bind to the LSU E site (LTM and cycloheximide (CHX))^45^, increased the proportion of ribosomes that efficiently carried out decoding by about twofold (Extended Data Fig. 10d,e). These observations support the existence of allosteric communication between leading and lagging edges of the human ribosome.

## Small molecules targeting mRNA decoding

**Fig. 5:**
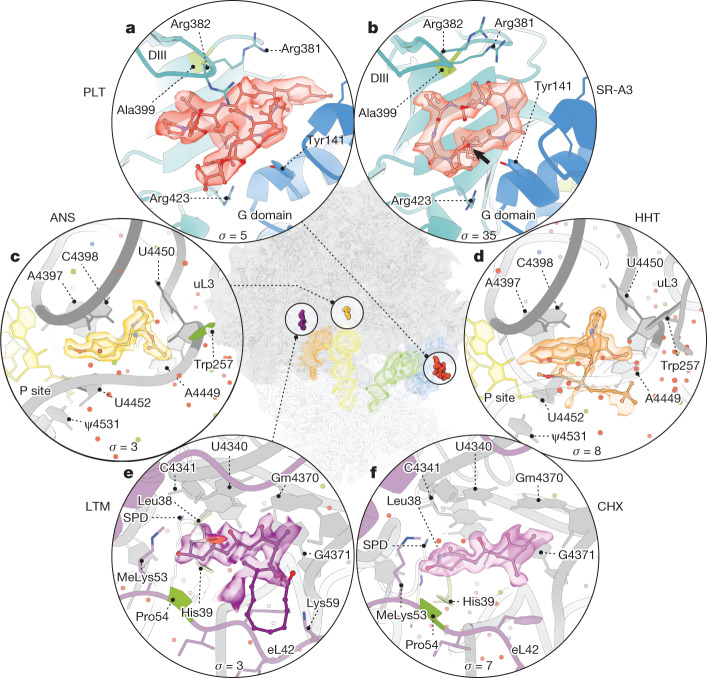
Binding sites for ribosome inhibitors. **a**,**b**, Overview of the binding sites for PLT (red) (**a**) and SR-A3 (coral) (**b**) on eEF1A between domain III (DIII, cyan) and the G domain (blue) bound to a GA-ribosome complex. Focus refined on eEF1A. The black arrow indicates the hydroxyl moiety that differentiates SR-A3 from ternatin-4. **c**–**e**, Structures of ANS (light orange) (**c**) and HHT (orange) (**d**) in the PTC and LTM (dark purple) (**e**) and cycloheximide (CHX, purple) (**f**) in the E site from consensus LSU focused refinements. The cryo-EM density from structures stalled with either PLT, ANS, LTM and GTPγS (**a**–**c**) or SR-A3, HHT, CHX and GTPγS (**d**–**f**) is shown. Known resistance mutations (green), waters (red), Mg^2+^ (lime green) and K^+^ (steel blue) are indicated. Contour levels for cryo-EM density are indicated in *σ* units.

In addition to the cryo-EM structures described above, we also solved consensus LSU and GA-complex ribosome structures stalled during decoding by GTPγS together with SR-A3, HHT and CHX (Extended Data Fig. 2 and Extended Data Table 1). Comparison of these structures with those stalled by GTPγS, PLT, ANS and LTM enabled us to compare the clinically relevant cyclic peptides PLT and SR-A3 bound to eEF1A and to assess the binding sites for CHX, LTM, HHT and ANS at sufficient resolution to resolve both solvent and ion contributions (Fig. 5).

PLT and SR-A3 bound to the G-domain-domain III interface of eEF1A at the same hydrophobic site as didemnin B and ternatin-4 (refs. ^24–26^), formed in part by collapse of the apical loop linking beta strands 5 and 6 in domain III towards the drugs (Fig. 5a,b). The buried surface area was larger for PLT than for SR-A3 (about 730 Å^2^ and about 560 Å^2^, respectively), correlating with their inhibitory effects (Fig. 1d and Extended Data Fig. 1f,g). As for didemnin B and ternatin-4 (refs. ^24–26^), PLT and SR-A3 adopted elongated folds supported by intramolecular hydrogen bonds (Fig. 5a,b). For both drugs, only a small set of non-ideal hydrogen bonds with eEF1A were evidenced. The increased potency and residence time of SR-A3 on eEF1A, which differs from ternatin-4 by a single hydroxy moeity^25^, suggests contributions to intramolecular ring stabilization, intermolecular hydrogen bonding potential to nearby Tyr141 in eEF1A or both.

As anticipated from structural investigations of vacant yeast^45^ and human^46^ ribosomes, ANS and HHT^27,28^ bound to a conserved tertiary fold within the PTC immediately opposite the 3′-CCA end of P-site tRNA, occluding the path of the nascent peptide into the exit tunnel (Fig. 5c,d). Both drugs stitched LSU rRNA U4452 (U2506 in *E. coli*) to their binding sites to disrupt stacking of Ψ4531 (U2585 in *E. coli*) on the aminoacylated terminus of P-site tRNA. Such changes are consistent with an altered induced-fit mechanism to prevent rapid peptide-bond formation after aa-tRNA accommodation^47^. The binding site for HHT was principally differentiated by its submarine-like extension, which pointed directly at the aminoacylated P-site tRNA terminus. The effect of this extension on the terminal adenosine of initiator tRNA and its ester linkage to the methionine amino acid probably explains its more efficient inhibition of proofreading selection and its specific inhibition of early translation steps before nascent peptide extension^28^.

Both LTM and CHX occupied a cavity in the E site directly overlapping with the binding site of the 3′-CCA end of deacyl-tRNA^45^, including elements of H74, the 2′OMe nucleotide G4370 and the methylated Lys53 residue of eL42, as well as neighbouring eL42 residues that confer resistance when mutated^48^ (Fig. 5e,f). Consistent with A-site/E-site allostery, the observed stabilization of the eIF5A–eL42 interactions correlated with Lys59 of eL42 forming contacts with the lactone ring of LTM in the AC structure (Extended Data Fig. 10c). No direct contacts with eIF5A were identified for either drug. As has been recently shown in *Neurospora*, the polar face of the glutarimide moieties of LTM and CHX hydrogen bonded with a highly ordered spermidine molecule wedged between the drug and residues Leu38 and His39 of uL15 (Fig. 5e,f), mutation of which confers cycloheximide resistance^48^. These data, which include local solvent geometries, are expected to aid efforts aimed at improving the clinical efficacies of each small molecule for the treatment of human disease.

## Discussion

The mechanism that enables the ribosome to rapidly decode mRNA using diverse aa-tRNA substrates defines the genetic code and is a paradigm of molecular recognition and movement in biology. How fidelity is established in translation and how signalling pathways and mutations in the translation machinery modify decoding fidelity in ageing and disease remain active areas of investigation^3,49^.

By combining multiperspective smFRET imaging and cryo-EM, we observe that the human ribosome—in cooperation with eEF1A—coordinates large-scale, sequential conformational changes within and between the ribosomal subunits to ‘read’ local geometric features of the L-shaped tRNA molecule with the SSU decoding centre and LSU GAC. Shape-associated distance measurements of the conserved tRNA geometry fundamentally underpin decoding fidelity during both initial selection and proofreading selection and appear to be universal, explaining the high degree of structural conservation of the ribosome core throughout evolution. In human, eukaryote- and potentially mammalian-specific elements of the ribosome and eEF1A contribute to decoding through molecular recognition events that are structurally and kinetically distinct from those that occur in bacteria to facilitate proper alignment of ternary complex and the aa-tRNA substrate with the catalytic centres of the ribosome. Structural elaborations of the human ribosome and eEF1A rationalize enhanced decoding fidelity in eukaryotic species. Key distinctions of the human decoding mechanism, established by eukaryote-specific components of the ribosome, the process of subunit rolling^18^ and the α2 helix in eEF1A, reduce the rate at which aa-tRNA enters the SSU (initial selection) and LSU (proofreading selection), providing more time for near- and non-cognate aa-tRNAs to dissociate.

Our findings suggest that conformational changes in the ribosome and eEF1A, together with physical properties of the incoming aa-tRNA molecule, constitute the most critical features by which decoding can be regulated. The eukaryote-specific α2 helix of eEF1A contributes to the decoding mechanism by directly interacting with evolutionarily distinct elements of intersubunit bridge B8, which remodels during both initial selection and proofreading selection. Notably, both bridge B8 elements and the α2 helix of eEF1A are targeted by diverse signalling pathways through post-translational modification^20,50^. Together with the proximity of post-transcriptional modifications to functional centres of both ribosomal subunits, we posit that conformational changes central to the decoding mechanism may be under regulatory control in eukaryotes^49^. Such features, together with the altered trajectories of tRNA motion, offer potential opportunities for species-specific—and context-specific—small-molecule intervention strategies. The allosteric linkage between the A and E sites associated with proofreading selection may further enable mammalian cells to assess and regulate the status of actively translating ribosomes. Conformational information of this kind, within both monosomes and polysomes, is likely to provide signals to other components of the cellular milieu that are central to protein synthesis regulation^22,51^.

## Methods

### Buffers and reagents

Human polymix buffer contained 20 mM HEPES pH 7.5, 140 mM KCl, 10 mM NH_4_Cl, 2 mM spermidine and 5 mM putrescine. Human polymix buffer was further supplemented with 5 mM MgCl_2_ and reducing agent (1 mM DTT for cryo-EM and 1.5 mM 2-mercaptoethanol for smFRET). Charge buffer contained 50 mM Tris pH 8.0, 10 mM KCl, 100 mM NH_4_Cl, 10 mM MgCl_2_, 1 mM DTT, 5 mM ATP and 0.5 mM EDTA. AM/FM buffer contained 50 mM Tris pH 7.5, 7 mM MgCl_2_, 150 mM KCl, 5 mM ATP, 0.5 mM DTT and 1 mM EDTA. Hybridization buffer contained 10 mm HEPES pH 7.0, 150 mm KCl and 0.5 mM EDTA. The above buffers were prepared as 5× stocks, flash-frozen in liquid nitrogen, stored at −80 °C and thawed just before use.

Ribosome lysis buffer contained 20 mm Tris pH 7.5, 100 mM KCl, 5 mM MgCl_2_, 1 mM DTT, 5 mM putrescine, 350 µM CHX, 4 U ml^−1^ RNase OUT, 1× HALT protease inhibitor, 0.5% NP-40 and 20 U of Turbo DNase. Subunit splitting buffer contained 20 mM Tris pH 7.5, 300 mM KCl, 2 mM MgCl_2_, 1 mM DTT and 2 mM puromycin. eIF5A buffer contained 50 mM phosphate buffer pH 8.0 and 300 mM NaCl. S buffer contained 50 mM Tris pH 7.0, 0.1 mM EDTA and 1 mM DTT. eIF5A storage buffer contained 25 mM HEPES pH 7.5, 100 mM KCl and 1 mM DTT. Acp labelling buffer contained 50 mM HEPES pH 7.5 and 10 mM MgCl_2_.

SR-A3 and PLT were synthesized in house; HHT was from Santa-Cruz biotechnology; and ANS, CHX and LTM were from EMD Millipore. All six drugs were dissolved in DMSO. GTP and GTPγS were from Sigma-Aldrich and were further purified using a Mono Q 5/50 GL column (Cytiva). Cy3-maleimide was synthesized in-house and LD555 and LD655-NHS were from Lumidyne technologies. DNA oligos and Gblocks were from IDT and synthetic RNA (mRNA) was from Horizon Discovery (Supplementary Table 2). Restriction enzymes (BamHI-HF and BpsDI), Cutsmart buffer and Gibson Assembly master mix were from NEB. DMEM, penicillin, streptomycin, trypsin-EDTA and PBS were from Lifetech, FBS was from Atlanta biologicals. Lipofectamine 2000, RNase OUT, Turbo DNase and DH10b cells were from Invitrogen. Quickextract was from Lucigen. Expi293 expression medium was from Gibco. Rabbit reticulocyte lysate was from Green Hectares. HALT protease inhibitor and HEK Expi293F cells were purchased from Thermo Fisher Scientific. BL21(DE3) pLys cells were from Sigma-Aldrich. All other chemicals were from Sigma-Aldrich or VWR.

### Generation of a cell line with A1-tagged uL11

We used CRISPR–Cas9-based genome engineering to generate cell lines expressing uL11 N-terminally fused to an A1 peptide tag^52^. HEK293T cells were grown to about 70% confluency in six-well plates in DMEM with 10% FBS and 100 µg ml^−1^ penicillin and streptomycin. Cells were then transfected with the plasmid PX459^53^ (Addgene, 62988) containing an sgRNA sequence targeting rpl12 (encoding uL11) near the start codon of the open reading frame (ORF), and an asymmetric dsDNA template^54^, for homology directed repair of the gene, using lipofectamine 2000. After transfection (24 h), cells were placed under puromycin selection (1.5 µg ml^−1^) for 48 h, exchanging the medium every 24 h. After selection, the cells were transferred to 10 cm culture dishes at a density of 1,000 cells per dish and left to grow until visible colonies formed. These colonies were transferred to 96-well plates using a pipette and grown to about 70% confluency. Each 96-well plate of cells was then split into two 96-well plates. After growth to around 70% confluency, one plate from each pair was screened for successful insertion of the A1 tag by lysing the cells in Quickextract and PCR amplifying the *rpl12* locus. PCR amplicons were treated with a restriction enzyme that cleaved modified, but not unmodified, alleles. After cleavage, the samples were loaded onto an agarose gel and the band pattern was analysed to identify unmodified, heterozygously modified and homozygously modified cell lines. Homozygous cell lines were propagated and used for purification of ribosomal subunits.

### Preparation of ribosome subunits

Ribosome subunits were prepared according to a protocol adapted from a previous study^19^. For wild-type subunits, HEK Expi293F cells were grown in Expi293 expression medium. When the cell density reached 6 × 10^6^ cells per ml, 350 µM CHX was added. Then, 5 min later, the cells were collected by centrifugation. For A1-uL11 subunits, HEK293T cells were grown in T225 flasks in DMEM with 10% FBS and 100 µg ml^−1^ penicillin and streptomycin to about 70% confluency, washed with PBS containing 350 µM CHX, lifted from the surface with 0.25% trypsin-EDTA containing 350 µM CHX, resuspended in DMEM with 350 µM CHX and pelleted by centrifugation. For both kinds of cells, the resulting pellets were resuspended in PBS buffer containing 350 µM CHX and pelleted again. Pellets containing around 300 million cells were flash-frozen in liquid nitrogen and stored at −80 °C.

To lyse the cells, pellets containing 1.8 × 10^9^ cells were placed in 50-ml stainless steel cryo-milling vessels (Retsch) and milled using a MM400 cryo-mill (Retsch) 5 times for 3 min at 25 Hz, with the vessels placed into a liquid nitrogen bath for 5 min between each milling cycle. The resulting cell powder was resuspended in ice-cold ribosome lysis buffer. The lysate was centrifuged at 4,000 rpm for 15 min at 4 °C using an Allegra X30R centrifuge (Beckman Coulter). The supernatant was then centrifuged again at 15,000 rpm for 15 min at 4 °C using a 5424-R centrifuge (Eppendorf). The supernatant from this second centrifugation was loaded onto six sucrose gradients (10–50% sucrose, 20 mm Tris pH 7.5, 100 mM KCl, 5 mM MgCl_2_, 1 mM DTT, 5 mM putrescine and 350 µM CHX) prepared using a Gradient Master IP 107 (Biocomp). The gradients were centrifuged at 30,000 rpm for 3 h at 4 °C using a SW32 rotor (Beckman Coulter) and the fractions containing polysomes were isolated using a BR-188 gradient analyser (Brandel). Polysomes were pelleted by centrifugation at 45,000 rpm for 18 h at 4 °C using a Ti45 rotor (Beckman Coulter).

To separate large and small ribosomal subunits (LSU and SSU, respectively), the polysome-containing pellets were resuspended in subunit-splitting buffer to a final volume of 2 ml and incubated at 37 °C with mild agitation for 30 min. Insoluble material was removed by centrifugation at 15,000 rpm for 15 min at 4 °C using the 5424-R centrifuge (Eppendorf) and the resulting supernatants were loaded onto six sucrose gradients (10–40% sucrose 20 mM Tris pH 7.5, 300 mM KCl, 2 mM MgCl_2_ and 1 M DTT) prepared using the Gradient Master IP 107 (Biocomp) system. The gradients were centrifuged at 40,000 rpm for 7 h at 4 °C using a SW41 rotor (Beckman Coulter) and SSU- and LSU-containing peaks were isolated using a BR-188 gradient analyser (Brandel). Ribosomal subunits were pelleted by centrifugation at 80,000 rpm for 12 h at 4 °C using a TLA100.3 rotor (Beckman Coulter). The ribosomal subunits (typically around 400 pmol) were resuspended in human polymix buffer containing 5 mM MgCl_2_ and 1 mM DTT, flash-frozen in liquid nitrogen and stored at −160 °C for future use.

### Preparation and labelling of tRNA^fMet^ and tRNA^Phe^

*E. coli* tRNA^fMet^ and tRNA^Phe^ were prepared and labelled with Cy3 and LD655, respectively, essentially as described previously^55,56^. *E. coli* tRNAs were used for reasons discussed previously^19^. A pBluescript plasmid for overexpression of tRNA^fMet^ was originally a gift from the laboratory of U. L. RajBhandary^57^. A pBluescript plasmid for overexpression of tRNA^Phe^ was originally a gift from the laboratory of K. Nierhaus^58^.

### Preparation of eEF1A and eIF5A1

Elongation factor eEF1A was isolated from rabbit reticulocyte lysate using a protocol modified from a previous study^59^, as described previously^19,40^.

Fully hypusinated eIF5A1 was prepared by overexpression in *E. coli* using the plasmid pST39 modified from a previous study^60^. The N terminus of eIF5A1 was extended by the addition of an A2 peptide tag^52^, a TEV cleavage site and a 6×His tag for purification. These modifications were inserted by first digesting the plasmid using BamHI-HF and BpsDI for 60 min in Cutsmart buffer removing the previous *EIF5A1* gene. A volume of the digestion reaction (7.2 µl) containing 140 ng of digested plasmid was then mixed with 10 µl of Gibson Assembly master mix and 0.4 pmol (6 µl) of a Gblock containing the modified *EIF5A1* sequence. The Gibson reaction was run for 30 min at 50 °C and then 2 µl of the mix was used to transform 25 µl of DH10b chemically competent cells.

For overexpression of eIF5A1, the modified pST39 plasmid was transformed into BL21(DE3) pLys cells, these were grown in LB medium at 37 °C containing 100 µg ml^−1^ ampicillin and 25 µg ml^−1^ chloramphenicol to an optical density at 600 nm of 0.5 when the temperature was adjusted to 18 °C and the cells were induced with 1 mM IPTG. Cells were collected 18 h after induction, flash-frozen in liquid nitrogen and stored at −80 °C. Cells (20 g) were resuspended in eIF5A buffer containing 10 mM imidazole and 1×HALT protease inhibitor and lysed by sonication using the Sonifier 450 (Branson) for 10 cycles of 45 s (duty cycle 6, intensity 60%) with 2 min on ice in between each cycle. The resulting lysate was cleared by centrifugation at 25,000 rpm for 30 min at 4 °C using a JA25 rotor (Beckman Coulter) and the supernatant was directly applied to a benchtop Ni-NTA column (Qiagen). After passage of the lysate, the column resin was twice resuspended in 10 ml of eIF5A buffer containing 20 mM imidazole. The bound eIF5A1 was eluted in 8 ml of eIF5A buffer containing 250 mM imidazole. The resulting eluate was dialysed (>250× dilution) overnight against S buffer. After dialysis, the eluate was diluted to 16 ml in S buffer, centrifuged at 4,000 rpm for 10 min at 4 °C using an Allegra X30R centrifuge (Beckman Coulter) and loaded onto a HiTrap SP HP column (Cytiva). eIF5A1 was then eluted using a 0–500 mM KCl gradient in S buffer, peaks representing hypusinated and non-hypusinated eIF5A1 (ref. ^60^) were collected separately and dialysed (>1,000× dilution) overnight against eIF5A storage buffer. eIF5A1 was flash-frozen in liquid nitrogen and stored at −160 °C for future use.

### Preparation of human ribosome ICs for smFRET

To prepare ribosome ICs containing P-site-bound Met-tRNA^fMet^ and displaying the codon UUC or UCU in the A site, mRNA was first annealed to a biotinylated dsDNA ‘pogo stick’ at its 5′ end by incubation of 100 pmol of mRNA with 100 pmol of dsDNA pogo stick in hybridization buffer at 37 °C for 5 min. Then, 20 pmol of SSU, heat-activated (42 °C for 5 min), was mixed with 80 pmol of mRNA pogo-stick complex in human polymix buffer in a total volume of 25 µl and incubated at 37 °C for 10 min. In parallel, 30 pmol *E. coli* tRNA^fMet^ was charged with methionine by incubation in AM/FM buffer containing 5 mM methionine and 6 µM MetRS for 15 min at 37 °C in a total volume of 10 µl. The charge reaction was then mixed with the mRNA-bound SSUs and incubated at 37 °C for 10 min. The resulting mixture was subsequently incubated with 20 pmol of heat-activated LSU for 20 min at 37 °C in a total volume of 50 µl. When fluorescently labelled LSUs were used, these were first labelled by incubation with CoA-activated LD555 and AcpS^61^ in Acp labelling buffer for 120 min at 37 °C. After incubation, the MgCl_2_ concentration was adjusted to 15 mM and the ribosome complex was purified by centrifugation for 90 min at 35,000 rpm in an SW41 rotor (Beckman Coulter) over a 10–40% sucrose gradient in human polymix containing 15 mM MgCl_2_ and 1 mM DTT, prepared using a Gradient Master IP 107 (Biocomp). The peak corresponding to the assembled ribosome complex was isolated off the gradient using a BR-188 gradient analyser (Brandel), flash-frozen in liquid nitrogen and stored at −160 °C for future use.

### Preparation of eEF1A ternary complex for smFRET

LD655-tRNA^Phe^ was charged with phenylalanine and bound into eEF1A ternary complex by incubation of 0.25 µM LD655-tRNA^Phe^ containing 0.1 µM eEF1A, 0.625 mM GTP or GTPγS, 3.75 mM phosphoenolpyruvate (PEP), 2.5 mM phenylalanine, 0.15 µM PheRS, 0.6 µM myokinase and 0.6 µM pyruvate kinase^41^ in charge buffer for 15 min at 37 °C. Ternary complex was stored on ice and used immediately after formation.

### smFRET data collection

All smFRET imaging experiments were performed at 25 °C or 37 °C using a custom-built, prism-type TIRF microscope^62^. Temperature control was achieved by enclosing the microscope stage in a temperature-controlled chamber. Surface-associated ribosomes were illuminated with a 532 nm diode pumped solid-state laser (Opus, LaserQuantum) at around 0.08 or 0.8 kW cm^−2^ (10-ms and 100-ms integration time, respectively). Fluorescence emission from donor and acceptor fluorophores was collected using a ×60/1.27 NA super-resolution water-immersion objective (Nikon), passed through a ET550lp filter (Chroma) to remove stray excitation light, spectrally split in a MultiCam Device (Cairn) with a 640lpxr dichroic filter (Chroma), passed through additional band-pass filters (ET585/65 and ET685/50, Chroma) and finally projected onto two aligned and synchronized ORCA-Fusion sCMOS cameras (C14440-20UP, Hamamatsu) with 2 × 2 pixel binning. Instrument control was performed using custom software written in LabVIEW (National Instruments). Donor and acceptor fluorescence intensities were extracted from the recorded videos, corrected for gamma and crosstalk and FRET efficiency traces were calculated using the SPARTAN software package^62^. FRET traces were selected for further analysis according to the following criteria: a single catastrophic photobleaching event, at least 8:1 signal/background-noise ratio and 6:1 signal/signal-noise ratio, less than four donor-fluorophore blinking events and a correlation coefficient between donor and acceptor of <0.5. The resulting smFRET traces were further analysed using HMM idealization methods, using the segmental *k*-means and MIL algorithms, as implemented in the SPARTAN software package.

### smFRET data analysis

All smFRET data were further analysed using the SPARTAN software package^62^ and standard nonlinear fitting and sorting methods implemented in MATLAB R2019a. Population histograms and transition density plots were generated in SPARTAN. Post-synchronization of traces to specific events was performed by first idealizing traces using HMM methods as implemented in SPARTAN, then identifying the first arrival in a state of interest. Traces were then aligned on the video frame corresponding to this event or to arrival in the first non-zero FRET efficiency state and truncated such that 5 frames before the event and 45 frames after the event remained. Post-synchronized population histograms and transition density plots were then generated to assess the dynamics surrounding the event of interest.

The ternary-complex association rate was estimated by identifying all events crossing the background noise threshold, eliminating the first and last events to minimize the influence of mixing time and photobleaching kinetics and then estimating the dwell times in the zero-FRET efficiency state between such events. These dwell times were then used to construct cumulative distributions for the zero-state dwell time. A single exponential function was fit to this distribution to estimate the mean zero-state dwell time, which was then used to calculate the association rate constant.

The catalytic efficiency of stable tRNA accommodation was estimated by identifying the first visit to the accommodated FRET efficiency state for each trace. Waiting times from the start of the trace to this event were then used to construct a cumulative distribution for the arrival time. A two-exponential function with a delay to account for the mixing time was fit to this distribution to estimate the mean arrival time, which was then used to calculate the catalytic efficiency.

The combined rate for all decoding steps after ternary complex binding was estimated by first identifying the first visit to the accommodated FRET efficiency state for each trace and then the first event passing the background noise threshold directly preceding this accommodation event. The times between these two events were then used to construct a cumulative distribution for the passage time. A two-exponential function was then fit to this distribution to estimate the mean passage time.

The equilibrium dissociation constant for eIF5A binding to the classical-state ribosome was determined by fitting of Gaussian functions to the equilibrium FRET efficiency distributions at different eIF5A concentrations to estimate the fraction of classical state ribosomes. These fractions were then plotted against the eIF5A concentration and the following equation describing equilibrium binding of a ligand to one of two possible binding partner conformations was fit to the data^63^:

$${f}_{{\rm{c}}}=\frac{{A}_{{\rm{C5A}}}\left({K}_{{\rm{D}}}+\left[{\rm{eIF5A1}}\right]\right)}{\left[{\rm{eIF5A1}}\right]+{K}_{{\rm{D}}}\frac{{A}_{{\rm{C5A}}}}{{A}_{{\rm{Capo}}}}}$$

Where *f*_c_ is the fraction of classical state ribosomes, *A*_C5A_ is the fraction of time the ribosome spends in classical-state-like FRET efficiency states when bound to eIF5A, *A*_Capo_ is the fraction of time the ribosome spends in classical-state-like FRET efficiency states when free from eIF5A and *K*_D_ is the dissociation constant for eIF5A binding to classical-state ribosomes.

### Preparation of ribosome complexes for cryo-EM

To prepare human ribosome ICs, 200 pmol of heat-activated (42 °C for 5 min) SSUs was mixed with 400 pmol of mRNA in human polymix buffer containing either 0 or 20 µM LTM in a total volume of 45 µl and incubated at 37 °C for 10 min. In parallel, 350 pmol *E. coli* tRNA^fMet^ was charged with methionine by incubation in AM/FM buffer containing 5 mM methionine and 6 µM MetRS for 15 min at 37 °C in a total volume of 12 µl. Complete tRNA charging was verified using a TSK phenyl-5PW column (TOSOH Bioscience). This charge reaction, containing 300 pmol of Met-tRNA^fMet^, was then mixed with the mRNA-containing SSU and incubated at 37 °C for 10 min. The resulting mixture was subsequently incubated with 200 pmol of heat-activated LSUs for 20 min at 37 °C in a total volume of 80 µl. After incubation, the MgCl_2_ concentration was adjusted to 15 mM and the ribosome complex was purified by centrifugation for 150 min at 35,000 rpm in an SW41 rotor (Beckman Coulter) over a 10–40% sucrose gradient in human polymix, containing 15 mM MgCl_2_, 1 mM DTT and either 0 or 20 µM LTM, prepared using a Gradient Master IP 107 (Biocomp). The peak corresponding to the assembled ribosome complex was isolated off the gradient using a BR-188 gradient analyser (Brandel), pelleted by centrifugation at 80,000 rpm in a TLA 100.3 rotor (Beckman Coulter) for 3 h and resuspended to a final concentration of 3.6 µM in human polymix buffer containing 5 mM MgCl_2_ and 1 mM DTT, flash-frozen in liquid nitrogen and stored at −160 °C for future use.

*E. coli* tRNA^Phe^ was charged with phenylalanine by incubation of a charge reaction containing 10 µM tRNA^Phe^, 2.5 mM phenylalanine, 3.75 mM PEP, 6 µM PheRS, 0.6 µM myokinase and 0.6 µM pyruvate kinase in charge buffer for 15 min at 37 °C. Complete charging of the tRNA was verified using the TSK phenyl-5PW column (TOSOH Bioscience). Ternary complex was then prepared by incubation of 0.55 µM eEF1A, 1 mM GTPγS, 40 µM eIF5A1 and enough charge reaction to bring the final Phe-tRNA^Phe^ concentration to 0.5 µM in human polymix buffer containing 5 mM MgCl_2_ and either 20 µM SR-A3, 50 µM HHT and 500 µM CHX, or 20 µM PLT, 50 µM ANS and 500 µM LTM for 10 min at 37 °C. After incubation, the ternary complex mixture was placed onto ice and immediately used for cryo-EM grid preparation.

Human ribosome ICs were mixed with eIF5A and either HHT and CHX or LTM and ANS and incubated for 60 s at room temperature before incubation with a ternary complex mixture yielding a final elongation complex mix containing 0.95 µM ICs, 0.25 µM ternary complex, 30 µM eIF5A and either 10 µM PLT, 50 µM ANS and 500 µM LTM, or 10 µM SR-A3, 50 µM HHT and 500 µM CHX.

### Cryo-EM grid preparation

Grids were prepared using a Vitrobot Mark IV plunge-freezing device (Thermo Fisher Scientific). For each experiment, immediately after ternary complex addition, 3 µl of the elongation complex mixture was applied to a QF R1.2/1.3 Au 300 mesh cryo-EM grid (Quantifoil) at 10 °C and 95% humidity that had been glow-discharged for 20 s in Ar/O_2_ using the Solarus II Plasma Cleaning System (Gatan). After addition of the elongation complex mix, the grids were immediately blotted (blot force −5) for either 14 s (PLT, ANS and LTM) or 6 s (SR-A3, HHT and CHX) and plunge-frozen into liquid ethane.

### Cryo-EM data collection instrumentation and procedures

Cryo-EM data were collected using the Titan Krios G3i (Thermo Fisher Scientific) transmission electron microscope operated at 300 kV accelerating voltage, equipped with a GATAN K3 direct electron detector operated in super-resolution mode and with a post-column BIO quantum GIF (energy filter). K3 gain references were acquired just before data collection. Data collection was performed using SerialEM software^64^ using image shift protocol (9 images were collected with one defocus measurement per 9 holes) at defocus values from −0.5 µm to −1.5 µm.

For the ribosome complexes bound to PLT, ANS, LTM, and GTPγS, videos were recorded with a magnification of ×105,000, which corresponds to a pixel size of 0.826 Å per pixel at the sample level (super-resolution pixel size was 0.413 Å per pixel). Data from three grids, collected during two sessions, were merged. Videos from grid 1 were collected with 70 frames (40 ms per frame) and a dose of 0.9401 e^−^ per Å^2^ per frame for a total dose of around 66 e^−^ per Å^2^. Videos from grid 2 were collected with 60 frames (50 ms per frame) and a dose of 1.318 e^−^ per Å^2^ per frame for a total dose of 79.101 e^−^ per Å^2^. Videos from grid 3 were collected with 60 frames (50 ms per frame) and a dose of 1.177 e^−^ per Å^2^ per frame for a total dose of 70.593 e^−^ per Å^2^.

For the ribosome complex bound to SR-A3, HHT, CHX and GTPγS, videos were recorded at a magnification of ×130,000, which corresponds to a pixel size of 0.6854 Å per pixel at the sample level (super-resolution pixel size is 0.3247 Å per pixel). During the 1.6-s exposure, 80 frames (20 ms per frame and the dose of 1 e^−^ per Å^2^ per frame) were collected with a total dose of around 80 e^−^ per Å^2^. Additional information on data collection parameters is provided in Extended Data Table 1.

### Cryo-EM data classification (PLT, ANS, LTM and GTPγS)

Motion correction was performed on raw super-resolution video stacks and binned twofold using MotionCor2 software^65^ separately for two data collections. CTF parameters were determined using CTFFind4^66^. Particles were picked using cisTEM^67^ and the coordinates were transferred to RELION (v.3.1)^68^ separately for two data collections. Particles from both data collections were pooled, extracted in in RELION (v.3.1) and particle stacks were transferred to cryoSPARC^69^. Several rounds of 2D classification using fourfold binned particles (pixel size = 3.304 Å per pixel) were performed to eliminate ice, carbon edges and false-positive particles. Particles were then imported and 3D auto-refined in RELION (v.3.1). All further classification was conducted in RELION (v.3.1).

Three global 3D classifications without alignment were run individually, varying the number of classes (*K*) and the regularization parameter (*T*). Classes containing eEF1A from independent classifications were combined and duplicate particles were removed. Similarly, classes containing classical ribosome particles without eEF1A were also pooled. One 3D classification also yielded a class containing P-site tRNA and eIF5A, which constituted the final accommodated reconstruction bound to eIF5A.

The set of particles containing classical ribosomes without strong evidence of eEF1A was 3D auto-refined (twofold binned). To identify as many eEF1A-bound particles as possible, we conducted four independent 3D classifications without alignment varying *K* and *T* parameters with soft masks around either (1) eEF1A only or (2) ‘ligands’: eEF1A, aa-tRNA and P-site tRNA. Both classifications using an eEF1A mask identified eEF1A-bound particles in two conformations (states I and II). Both classifications using a ligand mask identified particles containing state-I eEF1A. State I closely resembled the eEF1A-bound class identified in the first round of global classifications. All particles containing eEF1A in state I were pooled, duplicate particles were removed, remaining particles were 3D auto-refined and further classified to yield the GA reconstruction. Similarly, all particles containing eEF1A in state II were pooled, duplicate particles were removed, remaining particles were 3D auto-refined and further classified to yield the CR reconstruction. One of the classifications using a ligand mask also identified a class without eEF1A but containing P-site tRNA. This class was 3D auto-refined and was further classified to yield the IC reconstruction.

The 3D auto-refine map of the particles containing eEF1A in state II had comparatively weak cryo-EM density for eEF1A and aa-tRNA (ternary complex), so we conducted two successive rounds of focused 3D classification using soft masks around ternary complex, selecting particles with the strongest cryo-EM density for ternary complex. This class constituted the CR reconstruction.

We next aimed to identify particles containing E-site-bound eIF5A in the class without eEF1A and with eEF1A in state I using focused 3D classifications with a soft mask containing classical E-site tRNA and the L1 stalk. For particles containing eEF1A in state II, further classification on E-site content was avoided to preserve the particle number. For both sets of particles, we conducted three successive rounds of focused E-site classification to eliminate particles with E-site tRNA and select for particles containing eIF5A before 3D auto-refinement. For the set without eEF1A, the resulting class constituted the IC reconstruction bound to eIF5A. For the set containing eEF1A in state I and eIF5A, we observed heterogeneous density in which the SSU contacted eEF1A, so we conducted a focused 3D classification with a soft mask containing the SSU shoulder domain, selecting the class with strong density for eEF1A. This class constituted the GA reconstruction bound to eIF5A. Additional information on the cryo-EM classification pipeline and parameters is provided in Extended Data Fig. 2 and Extended Data Table 1.

To aid in modelling eEF1A and PLT, 131,115 particles identified as including eEF1A in state I were extracted from unbinned, polished particles and used for signal subtraction and 3D classification without alignment. This classification yielded 71,219 well-ordered particles that were then used for 3D auto-refinement and post-processing.

### Cryo-EM data high-resolution refinement (PLT, ANS, LTM and GTPγS)

Parallel to 3D classification of complexes, ribosome particles after the initial 3D classification step were pooled as indicated in Extended Data Fig. 2 (thick dotted grey line) and re-extracted without binning to obtain a consensus structure with focused refinement on the LSU. All of the steps involved in obtaining the consensus structure were performed in the beta 2 version of RELION (v.4.0)^70^. This consensus structure was used as input for CTF refinement to refine beam tilt, trefoil and tetrafoil aberrations, anisotropic magnification, per-particle defocus and per-micrograph astigmatism^68^. Optics groups (*n* = 27) were defined on the basis of image shift templates for each grid during data collection. Another LSU-focused refinement was performed before separating data from different grids/data collection sessions for Bayesian polishing^71^. After polishing, data were remerged for another LSU-focused refinement and CTF refinement was performed again to further refine parameters for anisotropic magnification, followed by beam tilt estimation and higher-order aberrations and finally per-particle defocus and per-micrograph astigmatism. Classes of interest that were determined during classification were selected from these ‘shiny’ particles for final rounds of 3D auto-refinement.

The consensus structure was also further processed for higher resolution and modelling purposes. Another LSU-focused refinement was performed on the merged particles after the second round of CTF refinement. An 80S refinement was also performed before an SSU-focused refinement with local angular searches. Finally, the metadata from the final refinements were used to create Ewald-sphere-corrected half-maps with relion_reconstruct for postprocessing to 1.67 Å for the LSU and 1.84 Å for the SSU. Sharpened and locally filtered maps were used for figure preparation and to aid in model building. Further information is provided in Extended Data Figs. 2 and 3 and Extended Data Table 1.

### Cryo-EM data processing procedures (SR-A3, HHT, CHX and GTPγS)

Motion correction was performed on raw super-resolution video stacks and binned twofold using MotionCor2 (ref. ^65^) separately for two data collections. CTF parameters were determined using CTFFind4 (ref. ^66^) and refined later in RELION (v.3.1)^68^ and RELION (v.4.0)^70^. Before particle picking, good micrographs were qualified by power spectrum. Particles were picked using cisTEM^67^ and the coordinates were transferred to RELION (v.3.1) separately for two data collections. The particles from both datasets were pooled, extracted in RELION (v.3.1) and particle stacks were transferred to cryoSPARC. Several rounds of 2D classification (fourfold and eightfold binned) were performed to eliminate ice, carbon edges and false-positive particles. Particles were 3D auto-refined (fourfold binned) in RELION (v.3.1) followed by two rounds of 3D classification—first with alignment (angular sampling interval of 1.8°, fourfold binned), then without alignment (twofold binned). Classes with density for eEF1A were selected for continued refinement (LSU consensus) or classification (GA).

For the LSU consensus complex, particles were extracted and refined at 0.685 Å per pixel, followed by 3D refinement with an LSU mask, CTF refinement, Bayesian polishing, 3D refinement with an LSU mask, CTF refinement and final 3D refinement with an LSU mask in RELION (v.4.0).

For the GA complex, another round of 3D classification in RELION (v.3.1) yielded a class with strong density for eEF1A. These particles were re-extracted from unbinned, polished particles generated in the LSU consensus complex processing and were auto-refined in RELION (v.4.0). Focused refinement of eEF1A with signal subtraction was also performed on these particles to aid with modelling of eEF1A and SR-A3. Sharpened and locally filtered maps were used for figure preparation and to aid in model building. Further information is provided in Extended Data Fig. 2 and Extended Data Table 1.

### Molecular model building

A human ribosome atomic model (Protein Data Bank: 6QZP) was manually fitted into the high-resolution (1.67 Å) consensus cryo-EM reconstruction using USCF Chimera^72^. Starting models were used to build into the cryo-EM map for tRNA^fmet^ (PDB: 3CW6), tRNA^Phe^ (PDB: 4WRO), eEF1A (PDB: 5LZS) and eIF5A (PDB: 3CPF). The mRNA was built de novo. Better agreement between the map and the model was achieved by group rigid body refinement, global minimization and simulated annealing refinement using phenix.real_space_refine^73^. Subsequently, ribosomal proteins and rRNAs were automatically built and refined using the ARP/wARP classic EM module in the CCP4 suite of programs^74^. The model geometry was further fine-tuned and agreement between the refined models and the cryo-EM maps was evaluated by map-model FSC according to a previously described method^75^ using CCP-EM^76^. The model was visually inspected together with the 3D volume and further improved by iterative model building in Coot^77^. As the ribosome with P-site tRNA was resolved at high resolution, post-translation and post-transcriptional modifications^78,79^ could be assigned unambiguously based on the experimental cryo-EM map. Furthermore, Mg^2+^ and K^+^ ions were assigned on the basis of coordination number and geometry^80^. The 3D models for small molecules, modified nucleotides and amino acids were built and the geometry restraints for model refinement were generated using JLigand^81^. This consensus atomic model was used as the initial model for the reconstructions of decoding intermediates obtained from the same dataset and the above-described protocol was then repeated for model building and refinement of each individual reconstruction. Additional information on model building procedures and statistics is provided in Extended Data Fig. 2 and Extended Data Table 1.

### Intersubunit bridge assignments

Intersubunit bridges were assigned as described previously^82^ (Extended Data Fig. 9a). For Fig. 4d and Extended Data Fig. 9, bridge contacts are considered to be formed when residing within 4 Å of each other. Each bridge contact point within the set of contact points composing an intersubunit bridge is assumed to contribute equally to the formation of that bridge. Total bridge formation (Extended Data Fig. 9b (percentage of bridge contacts)) represents the percentage of bridge contact points formed for each set of intersubunit bridge contacts. Change in bridge formation (Fig. 4d and Extended Data Fig. 9c (percentage change)) compares differences in individual contact points rather than changes in the total percentage of bridge contacts.

### Figure preparation

Molecular graphics and analyses were performed using UCSF Chimera^72^ and UCSF ChimeraX^83^. Cryo-EM map values were normalized for figure preparation to mean = 0 and *σ* = 1 in UCSF Chimera using the ‘vop scale’ function. Angle and distance measurements were performed in UCSF ChimeraX using the Fit in Map and the Distance tools, respectively. All of the figures were prepared using structures and models aligned to the LSU core, from a high-resolution human ribosome crystal structure (PDB: 6QZP) with the following mobile and peripheral elements omitted: LSU rRNA nucleotides: 747–914, 973–1279, 1429–1454, 1554–1569, 1696–1719, 1744–1781, 1956–2029, 2092–2263, 2476–2501, 2546–2593, 2649–2683, 2749–2770, 2895–3603, 3753–3774, 3944–4066, 4085–4164, 4241–4264, 4411–4427, 4753–4948 and 5007–5040; LSU ribosomal proteins: uL1, uL3, uL4 (amino acids 319–368), uL5, uL6, eL6, eL8, uL13 (amino acids 156–203), eL13, eL14, uL16, uL18, eL19 eL22, uL24, eL24, eL27, eL29, uL30, eL30, eL34, eL38, eL39, eL40, eL41, eL42 and eL43. Cryo-EM density is contoured at 3*σ* in all images, unless otherwise noted. r.m.s.d. heat maps were prepared in UCSF ChimeraX using the Matchmaker tool for proteins and nucleic acids. Electron density was coloured using the Color Zone tool in UCSF ChimeraX with a 3–5 Å radius. Figures were compiled in Adobe Illustrator (Adobe).

### Reporting summary

Further information on research design is available in the Nature Portfolio Reporting Summary linked to this article.

## Online content

Any methods, additional references, Nature Portfolio reporting summaries, source data, extended data, supplementary information, acknowledgements, peer review information; details of author contributions and competing interests; and statements of data and code availability are available at 10.1038/s41586-023-05908-w.

### Supplementary information

Supplementary Tables Supplementary Tables 1 and 2.

Reporting Summary

Supplementary Video 1 Overview of conformational changes during decoding seen from the head domain. Ribosomal conformational changes and ternary complex movements during mRNA decoding in human as seen from the SSU head domain. The video illustrates the minimal conformational changes after ternary complex binding in the IC to CR transition, SSU shoulder domain closure and eEF1A docking to the GAC in the CR to GA transition and SSU rolling and aa-tRNA accommodation in the GA to AC transition. SSU (light grey), LSU (dark grey), mRNA (pink), P-site tRNA (yellow), aa-tRNA (green) and eEF1A (blue) are indicated.

Supplementary Video 2 Overview of conformational changes during decoding seen from the leading edge. Ribosomal conformational changes and ternary complex movements during mRNA decoding in human as seen from the leading edge of the ribosome. The video illustrates the minimal conformational changes after ternary complex binding in the IC to CR transition, SSU shoulder domain closure and eEF1A docking to the GAC in the CR to GA transition and SSU rolling and aa-tRNA accommodation in the GA to AC transition. SSU (light grey), LSU (dark grey), mRNA (pink), P-site tRNA (yellow), aa-tRNA (green) and eEF1A (blue) are shown.

## Figures and Tables

**Extended Data Table 1:**
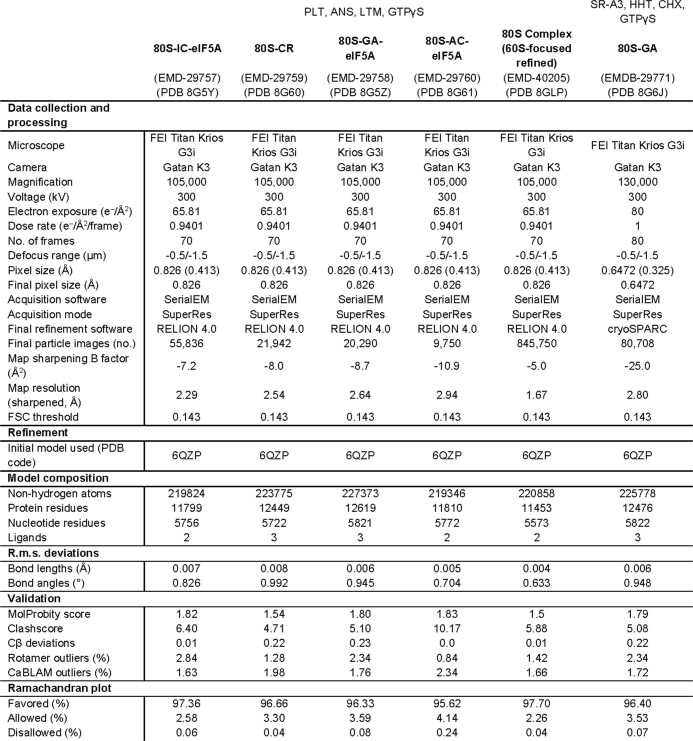
Grid preparation, data collection, refinement and model validation

## Data Availability

Cryo-EM maps and models were deposited at the Electron Microscopy Data Bank and RCSB Protein Data Bank, respectively, under the following accession codes: EMD-29757, 8G5Y (PLT, ANS, LTM and GTPγS-stalled IC-complex ribosome); EMD-29759, 8G60 (PLT, ANS, LTM and GTPγS-stalled CR-complex ribosome); EMD-29758, 8G5Z (PLT, ANS, LTM and GTPγS-stalled GA-complex ribosome); EMD-29760, 8G61 (PLT, ANS, LTM and GTPγS-stalled AC-complex ribosome); EMD-29771, 8G6J (SR-A3, HHT, CHX and GTPγS-stalled GA-complex ribosome); EMD-40205, 8GLP (PLT, ANS, LTM and GTPγS-stalled 80S complex 60S focus refined map); and EMD-29782 (SR-A3, HHT, CHX and GTPγS-stalled complex consensus refined map).

## Code availability

The software used for smFRET data analysis is freely available online (https://www.scottcblanchardlab.com/spartan-download).
