# Supplementary material for: mRNA decoding in human is kinetically and structurally distinct from bacteria
Source: Nature. 2023 Apr 5;617(7959):200–7. doi: 10.1038/s41586-023-05908-w (PMC10156603; doi:10.1038/s41586-023-05908-w)
Supplement: Supplementary file 1 — Supplementary Tables 1 and 2. [file 41586_2023_5908_MOESM1_ESM.pdf]

nature portfolio

https://doi.org/10.1038/s41586-023-05908-w

Supplementary information

mRNA decoding in human is kinetically and structurally distinct from bacteria

In the format provided by the authors and unedited

Supplementary Tables

Supplementary Table 1 | Estimated rate constants and mean times

Rate constant or mean time	Value
CR state lifetime (25 °C)	< 10 ms
GA state lifetime (25 °C)	620 ± 40 ms
Ternary complex association rate constant (25 °C)	70 ± 6 μM^{-1} s^{-1}
mRNA decoding catalytic efficiency (25 °C)	43 ± 3 μM^{-1} s^{-1}
mRNA decoding rate constant (25 °C)	1.7 ± 0.2 s^{-1}
mRNA decoding rate constant (37 °C)	12.8 ± 2.7 s^{-1}

Supplementary Table 2 | Nucleic acid reagent sequences.

Name	Sequence
sgRNA oligo 1	5’-CACCGTCGAACTTCGGCGGCATGG-3’
sgRNA oligo 2	5’-AAACCCATGCCGCCGAAGTTCGAC-3’
uL11 HDR template (double stranded)	5’-CGGGTTCATCCGACACCAGCCGC<br>CTCCACCATGGGCGACAGCCTGG<br>ACATGCTGGAGTGGAGCCTGATG<br>CCGCCGAAGTTCGACCCCAACGA<br>GATCAAAGTCGGTGCGTGCTCTG<br>GTTGTGGCCGGGGCTGCGG-3’
uL11 PCR primer 1	5’-CACCCTTCTCAGACTTCTCCGG-3’
uL11 PCR primer 2	5’-ACCCTCTGCCTCTTCTCGAAAC-3’
eIF5A1 gblock	5’-TAGGTGTTGGCACCATGCATCGCG<br>CCCGCGAAATTAATACGACTCACTA<br>TAGGGAGACCACAACGGTTTCCCT<br>CTAGAAATAATTTTGTTTAACTTTAA<br>GAAGGAGATATACATATGCATCATC<br>ACCATCACCACGAAAACCTGTATTT<br>TCAGGGCGACAGCCTGGACATGCT<br>GGAGTGGAGCGCAGATGACTTGGA<br>CTTCGAGACAGGAGATGCAGGGGC<br>CTCAGCCACCTTCCCAATGCAGTG<br>CTCAGCATTACGTAAGAATGGCTTT<br>GTGGTGCTCAAAGGCCGGCCATGT<br>AAGATCGTCGAGATGTCTACTTCGA<br>AGACTGGCAAGCACGGCCACGCCA<br>AGGTCCATCTGGTTGGTATTGACAT<br>CTTTACTGGGAAGAAATATGAAGAT<br>ATCTGCCCGTCAACTCATAATATGG<br>ATGTCCCCAACATCAAAAGGAATGA<br>CTTCCAGCTGATTGGCATCCAGGAT<br>GGGTACCTATCACTGCTCCAGGACA<br>GCGGGGAGGTACGAGAGGACCTTC<br>GTCTCCCTGAGGGAGACCTTGGCAA<br>GGAGATTGAGCAGAAGTACGACTGT<br>GGAGAAGAGATCCTGATCACGGTGC<br>TGTCTGCCATGACAGAGGAGGCAGC<br>TGTTGCAATCAAGGCCATGGCAAAAT<br>AAGTATCCGGGCCCTGTACAAGAT-3’
Pogo stick oligo 1	5’-GTAAGTTTTAGGTTGCCCCCCTTTTTT<br>TTTTTTTTTTTTTTTTTTTTTTTT-3’-Biotin
Pogo stick oligo 2	5’-AAAAAAAAAAAAAAAAAAAAAAAAAAA<br>AA-3’
mRNA UUC	5’-CAACCUAAAACUUACACACCCUUA<br>GAGGGACAACGUAUGUUCAAAGU<br>CUUCAAAGUCAUC-3’
mRNA UCU	5’-CAACCUAAAACUUACACACCCUUA<br>GAGGGACAACGUAUGUCUAAAGU<br>CUUCAAAGUCAUC-3’
