# Supplementary material for: mRNA decoding in human is kinetically and structurally distinct from bacteria
Source: Nature. 2023 Apr 5;617(7959):200–7. doi: 10.1038/s41586-023-05908-w (PMC10156603; doi:10.1038/s41586-023-05908-w)
Supplement: Supplementary file 2 — Reporting Summary [file 41586_2023_5908_MOESM2_ESM.pdf]

nature portfolio

Corresponding author(s): Scott C. Blanchard

Last updated by author(s): Feb 15, 2023

Reporting Summary

Nature Portfolio wishes to improve the reproducibility of the work that we publish. This form provides structure for consistency and transparency in reporting. For further information on Nature Portfolio policies, see our Editorial Policies and the Editorial Policy Checklist.

Statistics

For all statistical analyses, confirm that the following items are present in the figure legend, table legend, main text, or Methods section.

n/a	Confirmed	
☐	☒	The exact sample size (*n*) for each experimental group/condition, given as a discrete number and unit of measurement
☐	☒	A statement on whether measurements were taken from distinct samples or whether the same sample was measured repeatedly
☒	☐	The statistical test(s) used AND whether they are one- or two-sided<br>*Only common tests should be described solely by name; describe more complex techniques in the Methods section.*
☒	☐	A description of all covariates tested
☒	☐	A description of any assumptions or corrections, such as tests of normality and adjustment for multiple comparisons
☐	☒	A full description of the statistical parameters including central tendency (e.g. means) or other basic estimates (e.g. regression coefficient) AND variation (e.g. standard deviation) or associated estimates of uncertainty (e.g. confidence intervals)
☒	☐	For null hypothesis testing, the test statistic (e.g. *F*, *t*, *r*) with confidence intervals, effect sizes, degrees of freedom and *P* value noted<br>*Give P values as exact values whenever suitable.*
☒	☐	For Bayesian analysis, information on the choice of priors and Markov chain Monte Carlo settings
☒	☐	For hierarchical and complex designs, identification of the appropriate level for tests and full reporting of outcomes
☒	☐	Estimates of effect sizes (e.g. Cohen's *d*, Pearson's *r*), indicating how they were calculated

Our web collection on statistics for biologists contains articles on many of the points above.

Software and code

Policy information about availability of computer code

Data collection	SerialEM 3.9beta5 was used for automated Cryo-EM data aquisition, custom software implemented in LabView was used for smFRET data aquisition.
Data analysis	Cryo-EM data was analyzed, and atomic models built and refined using RELION 3.1, RELION 4.0, CryoSPARC 3.3.2, cisTEM 1.0, ctffind 4.0, UCSF Chimera 1.16, UCSF ChimeraX 1.3, Coot 0.98, ARP/wARP 8.0, CCP-EM 1.6, Phenix 1.20.1, JLigand.<br><br>smFRET data was analyzed using SPARTAN 3.7.0 implemented in Matlab R2019a, and custom scripts implemented in Matlab R2019a, available on request.

For manuscripts utilizing custom algorithms or software that are central to the research but not yet described in published literature, software must be made available to editors and reviewers. We strongly encourage code deposition in a community repository (e.g. GitHub). See the Nature Portfolio guidelines for submitting code & software for further information.

Data

Policy information about availability of data

All manuscripts must include a data availability statement. This statement should provide the following information, where applicable:

- Accession codes, unique identifiers, or web links for publicly available datasets
- A description of any restrictions on data availability
- For clinical datasets or third party data, please ensure that the statement adheres to our policy

Cryo-EM maps and models were deposited in the EMDataBank and RCSB Protein Data Bank (PLT/ANS/LTM/GTPγS-stalled IC-complex ribosome, EMD-29757, PDB-ID:8G5Y; PLT/ANS/LTM/GTPγS-stalled CR-complex ribosome, EMD-29759, PDB-ID:8G60; PLT/ANS/LTM/GTPγS-stalled GA-complex ribosome, EMD-29758, PDB-ID:8G5Z; PLT/ANS/LTM/GTPγS-stalled AC-complex ribosome, EMD-29760, PDB-ID:8G61; SR-A3/HHT/CHX/GTPγS-stalled GA-complex ribosome, EMD-29771, PDB-ID:8G6J. PLT/ANS/LTM/GTPγS-stalled complex 60S focus refined map, EMD-29766; PLT/ANS/LTM/GTPγS-stalled complex 40S focus refined map, EMD-29768; SR-A3/HHT/CHX/GTPγS-stalled complex consensus 60S refined map EMDB-29782.

All single-molecule data is represented in the manuscript. There is no standard repository for raw smFRET data and the combined size of the raw data files is large, ~3 TB. The raw data is available on request to SCB.

Human research participants

Policy information about studies involving human research participants and Sex and Gender in Research.

Reporting on sex and gender	*Use the terms sex (biological attribute) and gender (shaped by social and cultural circumstances) carefully in order to avoid confusing both terms. Indicate if findings apply to only one sex or gender; describe whether sex and gender were considered in study design whether sex and/or gender was determined based on self-reporting or assigned and methods used. Provide in the source data disaggregated sex and gender data where this information has been collected, and consent has been obtained for sharing of individual-level data; provide overall numbers in this Reporting Summary. Please state if this information has not been collected. Report sex- and gender-based analyses where performed, justify reasons for lack of sex- and gender-based analysis.*
Population characteristics	*Describe the covariate-relevant population characteristics of the human research participants (e.g. age, genotypic information, past and current diagnosis and treatment categories). If you filled out the behavioural & social sciences study design questions and have nothing to add here, write "See above."*
Recruitment	*Describe how participants were recruited. Outline any potential self-selection bias or other biases that may be present and how these are likely to impact results.*
Ethics oversight	*Identify the organization(s) that approved the study protocol.*

Note that full information on the approval of the study protocol must also be provided in the manuscript.

Field-specific reporting

Please select the one below that is the best fit for your research. If you are not sure, read the appropriate sections before making your selection.

☒ Life sciences ☐ Behavioural & social sciences ☐ Ecological, evolutionary & environmental sciences

For a reference copy of the document with all sections, see nature.com/documents/nr-reporting-summary-flat.pdf

Life sciences study design

All studies must disclose on these points even when the disclosure is negative.

Sample size	We did not perform statistical analysis to predetermine sample size. No statistical methods were used to predetermine sample size. All quantitative experiments were carried out in triplicate and means and standard deviations were calculated. All replicates were included in the analysis. The number of micrographs collected was determined by the number of particles required to achieve a high resolution reconstruction of the target complex.
Data exclusions	Raw smFRET traces were excluded from analysis if they did not accord to the following criteria: a single catastrophic photobleaching event, at least 8:1 signal/background-noise ratio and 6:1 signal/signal-noise ratio, less than four donor-fluorophore blinking events and a correlation coefficient between donor and acceptor < 0.5. Cryo-EM data analysis and classification can be found in Methods, Extended Data Fig. 2, and Extended Data Table 1.
Replication	All quantitative measurements were carried out in triplicate and all repeats were included in the analysis, when applicable. Cryo-EM data was processed according to standard methods and multiple datasets were collected yielding the same results.
Randomization	The experiments were not randomized as it is not applicable to structural determination and dynamics studies.

Blinding	The investigators were not blinded to allocation during experiments and outcome assessment as none of the experiments involved either human or animal models or group allocation.

Reporting for specific materials, systems and methods

We require information from authors about some types of materials, experimental systems and methods used in many studies. Here, indicate whether each material, system or method listed is relevant to your study. If you are not sure if a list item applies to your research, read the appropriate section before selecting a response.

Materials & experimental systems

n/a	Involved in the study
☒	☐ Antibodies
☐	☒ Eukaryotic cell lines
☒	☐ Palaeontology and archaeology
☒	☐ Animals and other organisms
☒	☐ Clinical data
☒	☐ Dual use research of concern

Methods

n/a	Involved in the study
☒	☐ ChIP-seq
☒	☐ Flow cytometry
☒	☐ MRI-based neuroimaging

Eukaryotic cell lines

Policy information about cell lines and Sex and Gender in Research

Cell line source(s)	HEK expi293 expi cells were from Thermo Fischer. HEK293T cells were from ATCC.
Authentication	HEK 293 expi was obtained directly from Thermo Fischer, HEK293T cell lines were authenticated by STR profiling.
Mycoplasma contamination	Cell lines tested negative for Mycoplasma.
Commonly misidentified lines (See ICLAC register)	No commonly misidentified cell lines were used.
